# Redox Homeostasis, Metabolic Pathways and Plasticity in Uveal Melanoma Compared to Other Cancers

**DOI:** 10.3390/cancers18152402

**Published:** 2026-07-25

**Authors:** Mihai Adrian Păsărică, Paul Filip Curcă, Christiana Diana Maria Dragosloveanu, Cosmin Ionuț Nisipașu, George Cristian Curcă

**Affiliations:** 1 Department of Ophthalmology, Clinical Institute for Ophthalmological Emergencies “Prof. Dr. Mircea Olteanu”, 010464 Bucharest, Romania; m.pasarica@yahoo.com (M.A.P.); christianacelea@gmail.com (C.D.M.D.); 2 Department of Ophthalmology, Central Military Emergency University Hospital “Dr. Carol Davila”, Carol Davila University of Medicine and Pharmacy, 010825 Bucharest, Romania; 3 Department of Ophthalmology, Clinical Institute for Ophthalmological Emergencies “Prof. Dr. Mircea Olteanu”, Carol Davila University of Medicine and Pharmacy, 010464 Bucharest, Romania; 4 Department of Dental Medicine I, Implant-Prosthetic Therapy, Carol Davila University of Medicine and Pharmacy, 020021 Bucharest, Romania; cosmin.nisipasu@gmail.com; 5 Department of Legal Medicine and Bioethics, Carol Davila University of Medicine and Pharmacy, 050474 Bucharest, Romania; cgcurca@yahoo.com

**Keywords:** uveal melanoma, redox homeostasis, redox signaling, antioxidants, NRF2, AMPK, mTOR, MAPK, FoxO, metabolic plasticity, cancers

## Abstract

**Background/Objectives**: To date there is a lack of an integrative review literature on redox homeostasis, oxidative stress and the influence on metabolic pathways and plasticity in uveal melanoma (UM), since most literature findings are from cutaneous melanoma (CM), a genetically and behaviorally distinct cancer. Furthermore, UM metabolic pathway comparison to other cancers could provide more insight into metastatic UM, a difficult-to-treat malignancy. **Methods**: A wide-ranging multi-step literature search of PubMed and Web of Science for redox balance, oxidative stress, antioxidants and metabolic plasticity in UM, with expanded search terms for connections with other cancers. **Results**: UM cells maintain redox homeostasis via several redox loops: glutathione, thioredoxin, peroxiredoxins, peroxisomal catalase and the mitochondrial antioxidative network. NADPH plays a key role in regenerating UM antioxidative capabilities. Key redox signaling pathways are the subject of ongoing research in UM: NRF2 signaling, AMPK, mTOR, MAPK, FoxO. These pathways are less studied versus CM and present behavior differences in UM. PON2, studied in CM, represents a literature gap in UM. Inside the tumoral microenvironment, UM presents high metabolic plasticity and easy switching from glycolysis to oxidative phosphorylation (OXPHOS). Thus, UM eschews the classic Warburg effect loop and instead presents high oxidative phosphorylation (OXPHOS) gene expression, which generates additional lactate, which in turn produces cascade reprogramming in the metabolic pathways and lactate metabolism particularities associated in experimental studies with immune-escape phenomena. Uveal melanoma’s OXPHOS capabilities confer survival advantages and subdivide tumoral populations into OXPHOS-high and OXPHOS-low variants. Glycolysis/OXPHOS metabolic plasticity is an ongoing research field in other cancers with common and different elements vs. UM: cutaneous melanoma, small cell lung carcinoma, pancreatic cancer, breast cancer, acute myeloid leukemia, prostate cancer, renal cell carcinoma and glioblastoma. Uveal melanoma cells are susceptible to deleterious effects of prooxidants, a metabolic vulnerability which helps to create genetic pleomorphism, selecting higher proliferation and dissemination variants. **Conclusions**: Uveal melanoma is an oncogenic mutation and mitochondrial metabolism-driven malignancy, with metabolic connections to other malignancies. Emerging understanding of redox homeostasis, redox pathway signaling, mitochondrial oxidative and oncogenic metabolism could lead to better understanding of therapeutic response and new therapeutic targets. This review novelly integrates the general and CM redox literature with the UM literature, painting a complex redox signaling and metabolic plasticity picture of UM.

## Simple Summary

Uveal melanoma is an intraocular malignancy that is particularized by effective primary tumor treatment (removing the tumor for which the patient presented), but poor long-term prognosis due to a high percentage of patients experiencing recurrent metastatic disease years after the primary tumor was successfully treated. This paper discusses in detail redox homeostasis and tumoral microenvironment features that reprogram glucose, fatty acid and oxidative metabolism in uveal melanoma and draws comparison to similar research ongoing in other cancers. Research shows that tumoral cells in uveal melanoma are highly adaptive, switching metabolic pathways to survive treatment and outcompete other tumoral populations. Uveal melanoma can switch from glucose metabolism to oxidative phosphorylation, increasing survival odds for the tumoral cells. These metabolic particularities could help better understand the molecular mechanisms for developing metastatic disease in uveal melanoma, as well as offer potential for new therapeutic targets for metastatic uveal melanoma.

## 1. Introduction

Uveal melanoma (UM) is the most commonly encountered primary intraocular cancer arising in adults [1] and represents 3–5% of all reported melanomas [2]. The most common sites for development of UM are choroidal melanocytes (85–90%), ciliary body (5–8%) and iris tissue (3–5%) [2]. From an epidemiology standpoint, males have a reported incidence 30% greater than females [2]. According to Krantz. B. et al., the median age of diagnosis for UM is around 62 years with the peak range between 70 and 79 years old [2]. The reported age range for UM is similar to cutaneous melanoma (greatest reported incidence >70 years old and greater in males) [3]. However, from a clinical and genetic standpoint, UM is very different from cutaneous melanoma (CM).

UM cells characteristically carry activating mutations in G-protein-coupled receptor (GPCR) signaling such as GNAQ and GNA11 component mutations in the alpha subunits of guanine nucleotide-binding proteins (G-proteins) Gα_q_ and Gα_11_ [4]. GNAQ and GNA11 gene mutations occur in a mutually exclusive manner in 93% of UM tumors [4,5] with GNAQ mutations detected in ~50% of UM tumors and GNA11 in ~43% of tumors [5]. These gene mutations effectively switch on the GTPase activity of G-proteins by turning on from an inactive state (bonded with guanosine diphosphate GDP) to an active state (bonded with guanosine triphosphate GTP) [4]. An important genetic event in the progression of UM to metastasis is inactivating somatic mutations in genes encoding the BRCA-1-associated protein 1 (BAP1) [6,7,8]. According to Singh N. et al., uveal melanoma is the most frequent tumor associated with BAP1 tumor predisposition syndrome (BAP1-TPDS) and furthermore can also manifest as bilateral primary uveal melanoma in BAP1-TPDS [8]. Mutations in BAP1 (80% of aggressive UM forms [4]) are associated with poor prognostic factors [5]. Other less common mutations than BAP1-reported mutations are Splicing Factor 3b Subunit 1 (SF3B1, exon 14, 26% of UM cases [9]) and Eukaryotic translation initiation factor 1A X-linked (EIF1AX, exons 1 and 2, 19% of UM cases [9]) [9]. Mutations in BAP1, SF3B1, EIF1AX are almost mutually exclusive with each other and may represent alternative downstream molecular events during tumor progression [5].

From a clinical standpoint, treatment of primary UM via surgery, radiotherapy or brachytherapy is often successful for the primary tumor; however, up to half of patients develop distant metastases [10] years after the original tumor was successfully treated [11]. UM characteristically presents a genetic liver-metastasis tropism which has been attributed by several authors to the tumoral expression of chemokine receptor 4 (CXCR4) [12,13] and C-C Chemokine Receptor Type 7 CCR7 [14]. Liver parenchyma presents high concentrations of CXCL12, the chemokine ligand for CXCR4 [14], while CCR7, also found in cutaneous melanoma, binds to the ligand CCL21 expressed in lymph nodes [14]. Overall reported metastatic rates by Longhitano L. et al. are 89% for the liver (84% of BAP1-mutated patients), 29% for the lung and 17% for bone metastases [6], with predicted survival at 15% upon 5 years [6]. Despite continuous advancement and research, the risk of metastasis remains high for uveal melanoma [15].

Oxidative stress is caused by the imbalance between the production and accumulation of oxygen reactive species (ROS) in cells and tissues [16]. According to Reddy V.P. et al., commonly encountered ROS are superoxide radicals (O_2_^•−^), hydrogen peroxide (H_2_O_2_), hydroxyl radicals (•OH), and singlet oxygen (^1^O_2_) [16]. Increased intracellular concentrations of ROS can lead to oxidative stress upon cellular structures such as proteins, lipids and nucleic acids [16,17]. Mitochondrial metabolism is the main contributor to physiological and pathological ROS production [16]. Other sources of O_2_^•−^ are cellular respiration, lipooxygenase (LOX) and cyclooxygenase (COX) activity during arachidonic acid metabolism and endothelial inflammatory cells [16]. Enzymatic cellular antioxidant defense systems are superoxide dismutase (SOD), catalase (CAT) and glutathione peroxidase (GPx) [16].

Research into the tumoral microenvironment of uveal melanoma (UM) cells paints a picture of adaptation for tumoral survival and progression [18]. According to Onken et al., selective inhibition of oncogenic Gq/11 signaling activates genetic cellular signals facilitating tumoral survival by increasing nutrient uptake and balancing redox homeostasis [18]. Furthermore, Blasi M.A. et al. noted that high proliferation rate tumoral cells present lower total antioxidant protection since they are less differentiated, in effect favoring a more mutagenic effect in the proliferative tumoral process [18]. Further research and parallels to other cancer types could offer more insight into the oncogenesis, proliferation and metastasis of UM.

## 2. Materials and Methods

A wide-ranging manual literature search was performed for the following repositories: Thomson Reuters Scientific Web of Science, Cochrane Library and The National Library of Medicine’s MEDLINE via PubMed. Firstly, articles regarding “melanoma” as in cutaneous melanoma, a genetically distinct cancer from uveal melanoma (UM), were excluded from the primary search.

**Figure 1:**
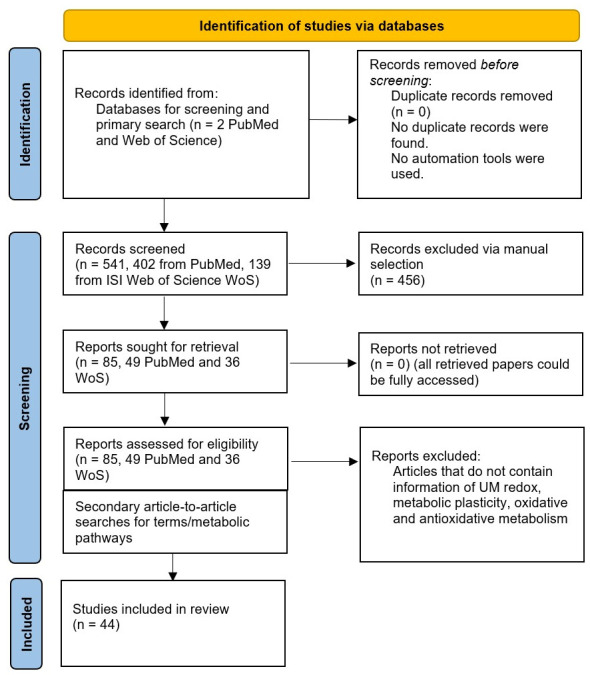
Summarized view of the primary conducted literature search. This flow diagram is adapted after the PRISMA 2020 guidelines presented by Page M.J. et al. [19] WoS = Web of Science.

Figure 1 presents a summarized view of the primary conducted literature search, adapted after The Preferred Reporting Items for Systematic Review and Meta-Analyses (PRISMA) statement [19].

The review was constructed through a primary search process (literature screening and selection) and a secondary search process which used terms/molecules/metabolic pathways from primary results to search article-to-article for additional information.

The primary literature screening search using the PubMed repository was performed manually in June 2026 by reviewer P.F.C. in collaboration with all paper authors focusing on results from the last 10 years. Use of the simple term “uveal melanoma” without any additional search terms yielded 402 results for the PubMed database. The results from PubMed were then manually screened by reviewer P.F.C. using title and abstract information for connections to UM redox metabolism, metabolic plasticity, oxidative and antioxidative activity. In total, only 49 papers were selected for the primary search results for extensive review by the paper authors (21 Antioxidant + 18 on Metabolic plasticity + 10 on Redox).

For the Web of Science (WoS), the primary literature screening search using the “Combined Semantic and Boolean” produced 3893 results after excluding ineligible publication types such as meeting abstract, editorial material, letter, correction, early access, book chapter, proceeding paper, retracted or expression of concern. Since the number of results, 3893, was too large to manually read and review, the WoS search was instead performed a second time using “Boolean” mode and combined search terms such as “uveal melanoma redox” (7 results), “uveal melanoma antioxidant” (32 results), “uveal melanoma metabolic” (98 results) and “uveal melanoma metabolic plasticity” (2 results). In total, 139 papers were selected for review by title and abstract. In total, 36 papers were extensively reviewed (27 from “uveal melanoma metabolic” and the rest from the other search terms).

The Cochrane Library was interrogated using the term “uveal melanoma”, returning 130 clinical trials in the last 5 years. Papers from The Cochrane Library were considered for inclusion if complementary to target pathways from the primary PubMed/WoS search. After careful consideration, we did not find noteworthy correspondence of data to papers in The Cochrane Library and thus papers from this source were not included in the final review.

To construct the review, we first selected relevant information from the primary search. Afterwards a secondary search was performed where information was searched article-from-article via target pathways/molecules stated in the primary search articles. Further information was gathered from article references on certain pathway topics. To search for comparison with other cancers, we searched by redox signaling and molecular pathways, finding articles in various other cancers and integrating the information.

A tertiary manual search was performed article-from-article on certain redox signaling topics that are incompletely understood in uveal melanoma. The authors note that uveal melanoma is less studied than cutaneous melanoma. Thus, when information for uveal melanoma, a genetically distinct cancer from cutaneous melanoma, was not available, the search continued into cutaneous melanoma articles and then general redox/biology articles. The authors have noted in-text information associated with cutaneous melanoma and general biology, information necessary especially for Section 3.1 and Section 3.2 of the manuscript. Thus, the tertiary search expanded molecular pathways into cutaneous melanoma and other applications, with information presented in a delimitated way versus uveal melanoma.

Table 1 summarizes the included studies from the primary and secondary searches.

## 3. Results

### 3.1. Endogenous Oxidative Pathways

Most literature findings on endogenous oxidative pathways are specified in connection with general cancer biology or cutaneous melanoma, a genetically distinct cancer from uveal melanoma. For explanation purposes, it is necessary to first describe the generalized concepts (sources of reactive oxygen, core endogenous antioxidant systems) and afterwards progress towards applications in uveal melanoma.

The most common reactive species encounterable in the TME redox system are superoxide radicals (O_2_^•−^), hydrogen peroxide (H_2_O_2_), hydroxyl radical (OH^–^), lipid peroxyl radicals (LOO•), lipid hydroperoxides superoxide radicals (LOOH) and singlet oxygen (^1^O_2_) [16]. Superoxide is produced when molecular oxygen accepts one electron [54] such as from electron leakage from the mitochondrial respiratory chain [54]. Other superoxide sources could be Nicotinamide Adenine Dinucleotide Phosphate (NADPH) Oxidases (NOX1-5) [55], xanthine oxidases [56], Cyclooxygenases (COXs) [56], lipid oxidase [56] and peroxisomes [57]. Superoxide does not diffuse freely and as such is reactive near the site where it is produced. Superoxide can be converted by superoxide dismutase into hydrogen peroxide [58]. Hydrogen peroxide can participate, in the presence of redox-active iron, in the Fenton reaction, producing a highly reactive and toxic hydroxyl radical [59] which can lead onwards to ferroptosis, an iron-sensitive subtype of regulated cell death [60]. Ferroptosis function is also underlined by Polyunsaturated fatty acids (PUFAs). PUFAs are part of cell membranes and are sensitive to oxygen. PUFAs can function as a substrate and rapidly react with oxygen, generating lipid peroxyl radicals and lipid hydroperoxides [61,62], triggering further processes in the ferroptosis pathway [62].

**Figure 2:**
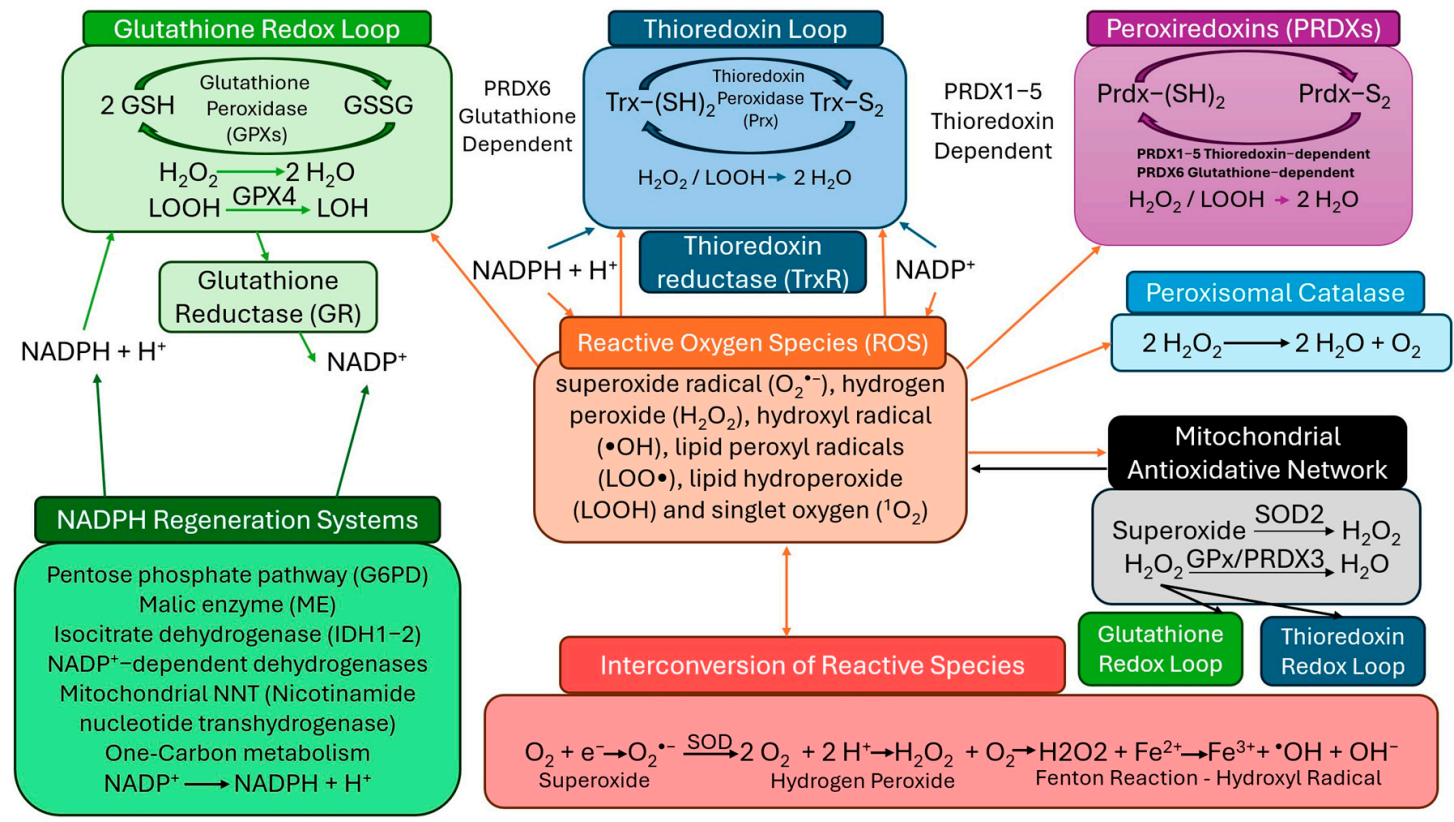
Hypothesized summary of potential redox homeostasis pathways in uveal melanoma using reviewed information from general redox biology, cutaneous and uveal melanoma data. This figure exemplifies avenues for detoxifying generated reactive oxygen species (ROS) for maintenance of redox homeostasis.

Generally speaking, there are several antioxidant loops and systems: superoxide dismutase, catalase, thioredoxin system, glutathione system and peroxiredoxins [20,63,64,65,66,67,68] (Figure 2). These antioxidant systems constitute an enzymatic defense against excess ROS. NADPH functions as a common reducing currency (fuel) which sustains the function of these antioxidant systems. NADPH regenerates the glutathione and thioredoxin systems, which in turn recycle glutathione peroxidases and peroxiredoxins to detoxify hydrogen peroxide and lipid hydroperoxides. NADPH activity is realized through four types of reactions: the pentose phosphate pathway, malic enzyme activity, isocitrate dehydrogenase and folate/one-carbon metabolism.

Superoxide dismutases (SODs) function as the first enzymatic defense against reactive oxygen species (ROS) through catalyzing the dismutation of superoxide (O_2_^•−^) into hydrogen peroxide (H_2_O_2_) and molecular oxygen (O_2_) [20,63]. Three isoforms maintain compartment-specific antioxidant protection: cytosolic SOD1 (Cu/Zn-SOD), mitochondrial SOD2 (Mn-SOD), and extracellular SOD3 (EC-SOD) [63]. Since hydrogen peroxide remains biologically reactive, SOD activity must be coupled with downstream peroxide-detoxifying enzymes (catalase, glutathione peroxidases (GPXs), and peroxiredoxins (PRDXs)) to prevent oxidative damage [20,63].

Catalase catalyzes the decomposition of hydrogen peroxide into water and oxygen. Thus, catalase represents one of the principal enzymatic mechanisms for eliminating excess intracellular H_2_O_2_ [64]. Catalase activity complements GPXs and PRDXs by preventing hydrogen peroxide accumulation following SOD-mediated superoxide dismutation [63,64].

The thioredoxin system consists of thioredoxin (TXN), thioredoxin reductase (TXNRD), and NADPH [64]. Oxidized thioredoxin is continuously regenerated by TXNRD using electrons supplied by NADPH, allowing reduced thioredoxin to repair oxidized protein thiols and regenerate peroxiredoxins during peroxide detoxification [65,66]. Consequently, the thioredoxin system plays a central role in maintaining intracellular redox homeostasis while cooperating closely with the glutathione system [65,66].

The glutathione (GSH) system represents the main low-molecular-weight thiol antioxidant system, essential for maintaining intracellular redox homeostasis [67]. Reduced glutathione (GSH) directly detoxifies hydrogen peroxide (H_2_O_2_) and lipid hydroperoxides (LOOH) through glutathione peroxidases (GPXs), producing oxidized glutathione (GSSG) [67,68]. Glutathione reductase (GSR) subsequently regenerates GSH from GSSG using reducing equivalents supplied by NADPH, thereby completing the glutathione redox cycle [67,68]. GSH also participates in protein thiol maintenance, iron homeostasis, and protection against ferroptosis through GPX4-mediated reduction of phospholipid hydroperoxides [62,68].

In general redox biology, the ascorbate–tocopherol cycle is a non-enzymatic antioxidant system that cooperatively protects both aqueous and lipid cellular compartments from oxidative injury [69,70]. During lipid peroxidation, α-tocopherol (vitamin E) donates a hydrogen atom to lipid peroxyl radicals (LOO•), forming the tocopheroxyl radical [69,70]. Reduced ascorbate (vitamin C) subsequently regenerates α-tocopherol. Oxidized ascorbate is recycled through glutathione-dependent reactions, linking this antioxidant cycle directly to the glutathione system [69,70]. To our knowledge, the exact function of the ascorbate–tocopherol cycle has not been mechanistically described in uveal melanoma. The authors have added this cycle due to its importance in general redox biology and state that there is a literature gap of knowledge specifically for uveal melanoma.

Peroxiredoxins (PRDXs) constitute a family of thiol-dependent peroxidases that catalyze the reduction of hydrogen peroxide, organic hydroperoxides, and peroxynitrite using reducing equivalents supplied primarily through the thioredoxin (TXN)–thioredoxin reductase (TXNRD) system [65,66]. During catalysis, the catalytic cysteine residue of PRDXs undergoes reversible oxidation, after which oxidized PRDXs are regenerated by reduced thioredoxin in an NADPH-dependent reaction [66]. This continuous recycling allows PRDXs to function as highly efficient peroxide scavengers while also participating in intracellular redox signaling [66].

The mitochondrial antioxidant network is a defensive antioxidant system against ROS generated during oxidative phosphorylation (OXPHOS) [71]. ROS are generated by electron leakage from respiratory chain Complexes I and III. The spare electrons combine with molecular oxygen [54] to generate superoxide, a potent ROS [71]. In response to the production of superoxide and to maintain redox homeostasis, mitochondrial SOD (SOD2) rapidly converts the produced superoxide into hydrogen peroxide [71]. Hydrogen peroxide is then detoxified by two interconnected antioxidant branches: the thioredoxin-peroxiredoxin system and the glutathione system [71,72,73,74]. The thioredoxin-peroxiredoxin system is primarily composed of the mitochondrial antioxidant enzymes Thioredoxin-dependent Peroxide Reductase (PRDX3), Peroxiredoxin 5 (PRDX5), Thioredoxin 2 (TXN2) and Thioredoxin Reductase 2 (TXNRD2) [71,72,73,74]. The glutathione system is composed of the mitochondrial antioxidant enzymes GPX1, GPX4, Glutathione Reductase (GR) and Glutathione (GSH). Both the thioredoxin-peroxiredoxin and glutathione mitochondrial antioxidant systems functionally depend on NADPH, which is regenerated within the mitochondrial matrix by Nicotinamide Adenine Dinucleotide Phosphate (NADP^+^)-dependent Isocitrate Dehydrogenase (IDH2), Malic Enzyme (ME3) and Nicotinamide Nucleotide Transhydrogenase (NNT) [71,72,73,74].

### 3.2. Redox Signaling

In general cancer biology, the Redox-activated Transcription Factor NF-E2-Related Factor 2 (NRF2) and Kelch-like ECH-associated Protein 1 (KEAP1) play an important role as a signaling master regulator [75,76]. The KEAP1-NRF2 pathway functions as a key regulator of cellular defenses against extrinsic and intrinsic oxidative and electrophilic stimuli [76]. According to Adinolfi, S. et al., key components of the KEAP1-NRF2 pathway are NRF2, KEAP1 and the CULLIN3 (CUL3) scaffold protein of the E3 ubiquitin ligase complex: CUL3 and KEAP1 form dimers, whereas only one NRF2 protein can be bound to a dimeric KEAP1 with two binding motifs, DLG and ETGE [76]. Somatic mutations of NRF2 and KEAP1 within the aforementioned binding sites are common in many cancers [76]. According to Carpenter E.L. et al., under normal conditions, there are low levels of NRF2 localized to the cytosol, where it is bound as sequestered by KEAP1 [77]. Exposure to oxidative stress redox-sensitive cysteine residues on KEAP1 triggers the release of NRF2 [77]. The literature data points towards NRF2 acting as a master transcription regulator in cutaneous melanoma [77,78]. Accumulated NRF2 enters the nucleus and activates oxidative stress-related genes associated with signaling pathways, immune infiltration and prognosis [77]. Carpenter, E. L et al. reported that a connection between NRF2 signaling and MAPK signaling in cutaneous melanoma [77]. Jessen C. et al. reported that knockdown of NRF2 in melanoma cell lines decreased proliferation and Epidermal Growth Factor Receptor (EGFR) expression [78].

NRF2’s role in uveal melanoma is an ongoing research domain. To our knowledge, there is not a full canonical description of the NRF2-KEAP1 pathway sequence in uveal melanoma. However, experimental data supports a role for NRF2-KEAP1 in uveal melanoma. Tonelotto, V.’s proteomic profiling data on the OMM2.5. UM cell line showed activation of the NRF2/Heme Oxygenase 1 (HO-1) axis after treatment with 1,4 dihydroxy quininib [21] and inhibition of GXP4 expression [21]. These biomarker modifications were associated with the ferroptosis pathway [21]. Furthermore, expression of genes inhibiting ferroptosis was significantly increased in UM patients with chromosome 3 monosomy, a known negative prognostic factor [21]. Barbosa, I. A. M. et al. reported the sensitivity of UM cells to knockdown of the Nuclear Receptor Subfamily 4 Group A Member 3 (NR4A3 or NOR1) and KEAP1 [79]. The Pan-cancer pathway analysis performed by The Cancer Genome Atlas (TCGA) did not highlight the NRF2-KEAP1 pathway as altered in uveal melanoma [80] and instead the most alteration in other cancers such as squamous lung cancer [80]. Thus, the findings of Tonelotto V. et al. support that NRF2 is involved in UM redox signaling; however, the extent of the importance of NRF2 is insufficiently studied to draw a conclusion.

Adenosin monophosphate (AMP)-activated protein kinase (AMPK) is an energy/ATP sensor composed of a catalytic α subunit encoded by α1 or α2, a scaffolding β subunit encoded by β1 or β2, and a regulatory γ subunit encoded by three γ genes (γ1, γ2 or γ3) [22,81]. According to Chua, V. et al., when AMP or Adenosin diphosphate (ADP) ratios decrease, AMP or ADP binds to the γ subunit of AMPK, promoting the phosphorylation of AMPK in the α subunit [22]. The activated AMPK promotes energy homeostasis by switching on catabolic processes and inhibiting anabolic pathways, thus reducing Adenosin triphosphate ATP consumption [22]. A secondary effect of AMPK is the activation of autophagy and protein degradation via lysosomes [22,82]. Chua V. et al. reported upregulation of the AMPK pathway in BAP1-mutant UM tumors [22]. Furthermore, they reported that BAP1 re-expression in UM cell lines MP46 and MP65 decreased pAMPK and pACC (Acetyl-CoA Carboxylase, ACC) levels, indicating BAP1-dependent regulation of AMPK signaling [22]. Deliktas, O. et al.’s cell culture data reported that the AMPK pathway represents a critical mechanism for mediating the survival of UM cells [23] with AMPK-inhibitors reducing the number of viable UM cells in culture (cytotoxic effect) [23]. Ambrosini, G. et al. reported that the AMPK pathway plays a role in selecting autophagic death over simple apoptosis of UM cells [24]. The authors reported that inhibition GNAQ signaling led to AMPK-dependent autophagic cell death [24]. Autophagy allows cellular repair processes and nutrient recycling and could be beneficial in theory to the UM TME, while apoptosis dismantles the cell [83]. Further experimental cell culture data from Proteau, S. supports that AMPK knockdown by small interfering ribonucleic acid (siRNA) reduces UM cell growth [25]. Wang J. Z. et al. reported early potential results in using AMPK activators to induce endoplasmic reticulum (ER) stress and autophagy on patient-derived UM xenografts [26]. The reported AMPK activator UC2288 could hypothetically provide an additional anti-metastatic effect by increasing expression of E-cadherin, stabilizing cell–cell adhesion and suppressing promigratory signaling [26]. A possible effect of AMPK activation is crosstalk interaction with Mammalian Target of Rapamycin Complex 1 (mTORC1), where there exists a negative feedback loop: in catabolism mTORC1 inhibits, AMPK activates while in anabolism mTORC1 activates, AMPK inhibits [84].

The Mechanistic Target of Rapamycin (mTOR) coordinates cell growth and metabolism with input nutrients and growth factors [85]. According to Saxton R.A. et al., mTOR is a serine/threonine protein kinase in the PI3K-related kinase (PIKK) family that forms the catalytic subunit of two distinct protein complexes: mTOR Complex 1 (mTORC1) and 2 (mTORC2) [85]. mTORC 1 is composed of three components: mTOR, Regulatory Protein Associated with mTOR (Raptor) and Mammalian Lethal with Sec13 Protein 8 (mLST8 also known as GβL) [85]. Raptor recruits substrate through binding to the TOR signaling (TOS) motif found on mTORC1 substrates [85], while in contrast, mLST8 associates with the catalytic domain of mTORC1 [85].

MAPK activation is characteristic of GNAQ and GNA11 mutations and less frequently of V600E BRAF mutations [27]. According to Ho A.L. et al., it has been hypothesized that activation of the PI3K/Akt/mTOR pathway cooperated with MAPK activation to generate and maintain malignant phenotypes in uveal melanoma [27]. This would be based upon the high rates of loss of heterozygosity at the Phosphatase and Tensin Homolog (PTEN) coding region coding a negative regulator of the PI3K/Akt/mTOR pathway [27]. Babchia N. et al. investigated whether inhibiting mTOR or PI3K-Akt signaling (Phosphatidylinositol 3-kinase PI3K, Protein Kinase B PKB also known as AKT) in cellular culture affects tumoral proliferation [28]. mTOR/Akt inhibition via rapamycin failed to inhibit proliferation of UM cells; furthermore, UM cells treated via mTOR inhibitor rapamycin exhibited increased Akt activation, with the authors concluding that UM cell lines are not sensitive to mTOR inhibitor rapamycin [28]. However, the same study reported that inhibition of PI3K reduced cell proliferation substantially and increased apoptosis [28]. This effect was observed regardless of B-Raf mutation status and attributed to inhibition of B-Raf/MAPK and PI3K signaling, but not mTOR signaling [86]. A wider-ranging study on seven cell lines by Amirouchene-Angelozzi reported that the mTOR pathway was shown to be activated in most of the cell lines independent of AKT signaling [29] and that inhibiting mTOR via Everolimus reduced the viability of UM cells in four of the seven cell lines [29]. A subsequent study led by the same author identified synergy between mTOR inhibitor Everolimus and PI3K inhibitor GDC0941 [30]. The authors reported that, mechanistically, it is most likely that Akt is reactivated by mTORC1 inhibition [30].

The Forkhead box O (FoxO) transcription factors are a direct downstream target of the PI3K/AKT pathway, comprising FoxO1, FoxO3a, FoxO4 and FoxO6 [31]. FoxO transcription factors are regulators of cellular proliferation, differentiation, DNA repair, oxidative stress defense, apoptosis and autophagy [31,87,88,89]. Yan F. et al.’s several studies examined FoxO3a in UM [32,33,90]. They studied in cell lines the role of FoxO3a in the Insulin Growth Factor 1 (IGF-1)-induced migration and invasion of UM cells [32]. Yan F. et al. reported that inhibition of FoxO3a via IGF-1 increased the viability, invasion and migration of UM cells [32]. IGF-1 increased the viability, invasion and migration of UM cells and it activated the IGR-1R/PI3K/Akt signaling pathway [32]. Mechanistically IGF-1 binds to its receptor and activates IGF-1R, which furthermore phosphorylates Akt, resulting in Akt translocations to the nucleus [32]. Akt interacts with FoxO3a in the nucleus and induces FoxO3a phosphorylation to regulate gene transcription [32,91,92]. Thus, FoxO3a is negatively regulated by the PI3K/Akt signaling pathway [32,93]. Inhibition of FoxO3a increases cellular invasion and migration of UM in cell lines, downregulating Bcl-2 Interacting Mediator of Cell Death (BIM) and p27 (also known as FoxO3) expression and upregulating Cyclin D1 expression [32,33]. In another paper, Yan F. et al. reported that FoxO3a overexpression increased the transcription and expression of Bcl-2-Like Protein 11 and Cyclin-dependent Kinase Inhibitor 1B and inhibited Cyclin D1 transcription and expression [33]. In a third study, Yan F. et al. linked pristimerin-induced apoptosis in UM cells (UM-1) to direct correlation with inhibition of the PI3K/Akt/FOX3 pathway [31,90] and postulated a possible cytotoxic effect of pristimerin on UM UM-1 cells [31,90]. Overall, FoxO3a activation acts as a brake for cellular invasivity which could potentially make FoxO3a a theoretical therapeutic target for UM tumor suppression [32,33,90].

Paraoxonase-2 (PON2) is a dual function lactonase enzyme that hydrolyzes bacterial signaling and furthermore presents a potent anti-oxidative effect, influencing redox signaling and cell survival [94]. PON2 specifically reduces superoxide released from the inner mitochondrial membrane, irrespective of whether it results from Complex I or Complex III of the electron transport chain [94]. PON2 has been found to be upregulated by several human tumors [95]. This is important since PON2 modulates execution of the cell death program directly at the mitochondria, allowing modification of several converging apoptotic pathways [95]. PON2 is a potential biomarker associated with overexpression in cutaneous melanoma and non-melanoma skin cancers (NMSCs) such as basal cell carcinoma (BCC) [96]. According to Brănişteanu, D. E. et al., PON2 expression correlates with important prognostic parameters [97]. Campagna, R. et al. reported that silencing PON2 enhanced the sensitivity of cutaneous melanoma to cisplatin [98]. Thus, silencing PON2 may enhance chemosensitivity, supporting it as a potential therapeutic target in redox adaptation and treatment resistance [95]. To our knowledge there is a lack of data on PON2 in uveal melanoma, a literature gap for future research.

### 3.3. Uveal Melanoma Tumoral Cells Present Genetic Heterogeneity and Continuous Oncogenetic Evolution

According to Durante M.A. et al., who used single-cell ribonucleic acid sequencing (scRNA-seq), sequenced 59,915 tumor and non-neoplastic cells from eight primary and three metastatic samples [34]; tumoral populations varied, with cellular complexity increasing from primary class 1 (BAP1 wild-type tumors) to class 2 (BAP1 mutant tumors) [34]. The authors further used T cells, which were present in all tumoral samples, to perform hierarchical clustering of the average gene expression per cluster across tumor samples [34]. Durante M.A. et al. firstly noted that previous studies showed that canonical genomic aberrations (oncogenetic driver events) arise early in uveal melanoma and give rise to one of three evolutionary trajectories: EIF1AX in class 1A, SF3B1 and other splicing mutations in class 1B and BAP1 in class 2 tumors [34]. Secondly, Durante M.A. et al. revealed through scRNA-seq sequencing that tumoral populations continue to further evolve continuously, developing non-canonical subclones that may contribute to tumor/disease progression [34]. Some of these mutations, as discussed by Durante M.A. et al., could be advantageous in eluding immune surveillance by creating an immunosuppressive microenvironment that promotes metastasis through immune escape [34]. Furthermore, the high mutation load could exhaust longer-term immune response [34].

### 3.4. Uveal Melanoma Adaptations to Hypoxia

Dong L. et al. highlighted the genetic adaptation of uveal melanoma to hypoxia via hypoxia inducible factor (HIF), a major transcriptional signature of specific subsets of human uveal melanoma [35]. In conditions of hypoxia of the tumoral microenvironment, HIF activation leads to gene expression that mediates metabolic adaptation to anaerobic growth, angiogenesis, invasion and metastasis [35,99,100]. Disruption of HIF-1α recruitment of p300/CBP cofactors could impair tumoral hypoxia adaptation as well as oncogenic signaling via the CXCR4 and c-Met receptor tyrosine kinase which can bind hepatocyte growth factor (HGF) [35]. This tumoral proliferation avenue is possibly linked to uveal melanoma propensity for liver metastasis via the CXCR-4 receptor pathway tropism [12,13,14,35].

Another research avenue for hypoxia and uveal melanoma was observing if hypoxia altered immune response in the presence of uveal melanoma cells. Bronkhorst, I. determined that exposure to hypoxia led to increased expression by uveal melanoma cells of several proinflammatory cytokines: Placental Growth Factor (PLGF, which is similar to Vascular Endothelial Growth Factor VEGF), Transforming Growth Factor Beta (TGFβ), Endothelin-1 (END1) and Intercellular Adhesion Molecule 1 (ICAM1) [36]. The authors noted lowered expression of Aminoacyl tRNA Synthetase Complex-Interacting Multifunctional Protein 1 (AIMP1), Chemokine Ligand 2 (CCL2, also known as Monocyte Chemoattractant Protein-1 MCP-1) and Interleukin-1-Beta (IL1β) [36]. Overall, the authors hypothesized that, under hypoxic conditions, uveal melanoma acquires an inflammatory phenotype and activates specific immune gene expression [36]. These changes may affect the immune response to the tumor by affecting the differentiation of peripheral blood monocytes inside the tumor into immunosuppressive and proangiogenic M2 macrophages [36]. Bronkhorst, I. et al. further hypothesize that hypoxia could be essential for infiltration and activation in vivo for the changes observed via Hypoxia Inducible Factor (HIF) [36], as previously discussed by Dong L. et al. [35]. Hypoxia-induced changes lead to upregulation and downregulation of inflammatory genes in primary uveal melanoma cultures, with VEGF as the most differentially expressed molecule [36]. Furthermore, a regulator gene for VEGF, the VHL gene, is located on chromosome 3 [36], and monosomy 3 is an important risk factor for poor prognosis of UM [37].

### 3.5. Lactate Metabolism

Longhitano L. et al. highlighted that lactate concentrations reprogrammed metabolic behavior of uveal melanoma suppressing tumoral growth [6], with lactate behaving as a signal molecule by Hydroxycarboxylic Acid Receptor 1 (HCAR1)-mediated cascade through the import channel Monocarboxylate Transporter 1 (MCT1) and export channel Monocarboxylate Transporter 4 (MCT4) [6]. Lactate supplementation boosts the expression of the transporters, and boosts Oxidative Phosphorylation (OXPHOS) activity [6]. The authors argued that lactate promotes a euchromatic state, characterized by enlarged nuclei and, therefore, quiescent cells display reduced proliferation, more relaxed chromatic and may rely more on oxidative phosphorylation over glycolysis [6].

Gong C. et al. investigated links between uveal melanoma lactate metabolism and Interleukin-6 (IL6) [38]. Previously, The Cancer Genome Atlas (TCGA) findings associated increased IL-6 messenger ribonucleic acid (mRNA) with tumor stage, correlating it with poor odds survival and lower disease-free survival times [38,39,101]. Experimental data showed that IL-6 exposure led to upregulation in Programmed Cell Death Ligand 1 (PD-L1) and Human Leukocyte Antigen E (HLA-E) levels, thus favoring immune escape [38]. Furthermore, IL-6 increased enzyme activity encoding Phosphofructokinase (PFK) and Pyruvate Kinase (PKM) in the glycolysis pathway, increased production of lactate and suppressed enzymes in the acetyl-CoA synthesis pathway [38]. This lactate-IL6-glycolysis feed-forward model is considerably experimental (experimentally demonstrated) and this review paper’s authors note that currently there is a lack of integrative studies on these findings in the clinical context of uveal melanoma.

### 3.6. Resistance to Oxidative Stress

Uveal melanoma exhibits metabolic reprogramming inside the tumoral microenvironment to resist oxidative stress. Wu, Y. et al. reported a 1.44 ± 0.012-fold overexpression of Calmodulin-1 (CALM1) in uveal melanoma samples treated with Hydrogen peroxide (H_2_O_2_) [40]. The authors induced oxidative stress via H_2_O_2_, which suppressed Superoxide Dismutase SOD and produced elevated concentrations of Malondialdehyde (MDA) and Lactate dehydrogenase (LDH) [40]; oeCALM1 effectively reversed measured oxidative stress by increasing SOD and suppressing accumulation of MDA and LDH [40]. Furthermore, the authors suggested that overexpression of CALM1 could influence CD4+ T-Cell response since exosomal regulation of Ca^2+^ signaling has been shown to reduce CD40 Ligand (CD40L) expression [40] and suppress CD4+ T-cell activation and proinflammatory cytokine secretion [40]. Ca^2+^ signaling is also involved in differentiating naive CD4+ T-cells into T-Helper-17 (Th17) and Treg subsets [40].

#### Calcium-ROS Axis in Uveal Melanoma

Tumoral infiltration with CD8+ T-Cells, macrophages and Natural Killer (NK) are associated with poor prognosis uveal melanoma [40,41,42], with immune scores lower in higher-risk groups [40]. Ca^2+^ signaling is regulatory to NK activity by maintaining homeostasis of secretory lysosomes and their interaction with mitochondria [40], with deletion of calcium channels in NK cells impeding autophagic capabilities, leading to accumulation of dysfunctional mitochondria and increased oxidative stress [40]. Endoplasmic reticulum (ER) calcium imbalance in cutaneous melanoma similarly triggers mitochondrial swelling, cell necrosis and apoptosis [102]. Ambudkar I. et al. described bidirectional interactions between reactive oxygen species (ROS) and Ca^2+^ signaling [103], wherein ROS can regulate cellular Ca^2+^ signaling, and Ca^2+^ signaling is essential for ROS production [103]. ROS affect almost all aspects of Ca^2+^ signaling such as the ER, cytosol, nucleus, mitochondria, lysosomes and plasma membrane [103].

### 3.7. Uveal Melanoma Oxidative Phosphorylation Genetic Profile Presents Genes Common to Other Cancers

Metastatic uveal melanoma heavily relies on mitochondrial oxidative phosphorylation (OXPHOS) [104] via the macroH2A1 histone variant [104] (loss of macroH2A1 decreases mitochondrial metabolism and reduces aggressivity of UM [43]) and eschews traditional glycolytic metabolism associated with other cancers [104]. This behavior is particularly distinct versus cutaneous melanoma where the presence of macroH2A isoforms is correlated with reduced aggressivity [43,105], and knockdown of mH2A isoforms in melanoma cells of low malignancy results in significantly increased growth and metastasis of cutaneous melanoma [43,105]. Zhan, Z. et al. identified a total of nine OXPHOS-related prognostic genes and constructed a prognostic risk model based on [44]: the NDUFB1 gene for encoding Nicotinamide Adenine Dinucleotide (NADH) Dehydrogenase (ubiquinone) 1-β Subcomplex Subunit 1, NDUFB8 gene for encoding NADH Dehydrogenase (ubiquinone) 1-β Subcomplex Subunit 8, ATP12A gene for encoding Potassium-transporting Adenosine-triphosphatase (ATPase) α-chain 2, NDUFA3 gene encoding NADH dehydrogenase (ubiquinone), CYC1 gene encoding The Respiratory Subunit of Ubiquinol Cytochrome C, COX6B1 gene encoding Cytochrome C Oxidase Subunit 6B1, ATP6V1G2 gene encoding a Component of Vacuolar ATPase, ATP4B gene encoding the glycosylated, non-catalytic β-Subunit of the H^+^/K^+^-ATPase proton pump, and NDUFB4 gene encoding NADH dehydrogenase (ubiquinone) 1-β-Subcomplex-4 [44]. The OXPHOS genes are connected with mitochondrial metabolism [44]. In general mitochondrial metabolism enhancement in OXPHOS activity increases electron flow through the electron transport chain [106,107] which can generate electron leakage that reacts with molecular oxygen to generate superoxide (O_2_^•−^) and other reactive oxygen species [106,107]. The CYC1-oncogene has prognostic influence on uveal melanoma [44]. Several of the eight novel OXPHOS genes identified in uveal melanoma by Zhan, Z. et al. (the ninth CYC1 was previously studied) have also been identified in other cancers [44]. NDUFB1 and NDUFA3 are associated with OXPHOS pathway alterations in clear-cell renal-cell carcinoma [44,108] and lung squamous cell carcinoma [44,109]. NDUFB4 has been associated with poor prognosis colorectal cancer [44,110] and gastric cancer [44,111]. Downregulated plasma ATP4B is a potential serum biomarker in gastric cancer [44,112]. The NDUFB8 gene can be hypermethylated in glioblastoma [44,113]. ATP6V1G2 of the V-ATPase is related to glioma prognosis [44,114]. Finally, ATP12A can be overexpressed in pancreatic cancer [44,115] and prostate cancer [116].

### 3.8. GNAQ and GNA11 Oncogenes and Redox Implications

Activating mutations in Guanine Nucleotide-Binding Protein G(q) subunit α (GNAQ) and Guanine Nucleotide-binding Protein Subunit α-11 (GNA11) occur in over 90% of uveal melanomas [45]. These α-subunits of the heterotrimeric G proteins Gq or G11 (Gq/11) are thus an oncogenic driver in almost all (90%) of uveal melanoma cases [18]. According to Elbatsh et al., GNAQ/GNA11 mutant UM cells produce high levels of the second messenger inositol 1,4,5 triphosphate (IP3) that accumulate upon suppression of Inositol-1,4,5-triphosphate 5-phosphatase (INPP5A) [45], resulting in hyperactivation of IP3-receptor signaling, increased cytosolic calcium and p53-dependent apoptosis [45,117]. p53 signaling has also been identified as an important player in cutaneous melanoma progression [118]; resistance to the apoptotic trigger promotes neoplastic cell survival and resistance [118].

Onken M.D. et al. reported that inhibition of the oncogenic Gq.11 with the small molecule FR900359 attenuated glucose uptake by UM cells in vivo and in vitro, blunted glycolysis and mitochondrial respiration and reduced levels of several glycolytic and tricarboxylic acid cycle intermediates [18]. The UM cells adapted in consequence by upregulating expression of genes involved in metabolite scavenging and redox homeostasis [18], contrary to the classic Warburg effect in cutaneous melanoma [18] (Warburg effect: increased glucose uptake and fermentation of glucose to lactate, leading to progressive acidification of the tumoral microenvironment [119]). It thus would seem that GNAQ/GNA11 metabolic reprogramming favors neoplastic cell survival by maintaining suitable redox homeostasis for tumoral populations.

Selectively targeting GNAQ/11 activity by increasing reactive oxygen species (ROS) production, such as described by Li Y. et al. using the copper ionophore elesclomol, leads to a signaling cascade that suppresses the migration of UM cells [46]. Li Y. et al. described the following effect cascade [46]: elesclomol transports copper to mitochondria which produce a large amount of ROS by reduction of Cu2 to Cu1 in GNAQ/11 mutant cells [46], selectively activating Large Tumor Suppressor Kinase 1 (LATS1) kinase, which promotes Yes-associated Protein (YAP) phosphorylation in the hippo pathway [46,120], inhibiting its nuclear accumulation, which in turn suppresses the migration of uveal melanoma cells [46]. Elesclomol’s effect was found to be sinergic with binimetinib, a Mitogen-Activated Protein Kinase (MEK) inhibitor [46]. MEK inhibitors have also been studied in other cancers such as melanoma and non-small cell lung cancer [121], with two MEK inhibitors approved for use in metastatic cutaneous melanoma by the United States Food and Drug Administration (FDA) and the European Medicines Agency (EMA): trametinib and cobimetinib [121]. A multitude of other MEK inhibitors are in clinical trials for a wide variety of cancers [121].

### 3.9. BRCA1-Associated Protein (BAP1) Mutations and Implications on Redox Metabolism

Breast Cancer 1, BRCA-associated protein (BAP1) is associated with metastasis in uveal melanoma [47]. According to Han A. et al., the BAP1 gene, located on chromosome 3p21.1 [47], is a tumor suppressor that acts as a deubiquitinating enzyme (DUB) in key cellular pathways such as the cell cycle, Deoxyribonucleic Acid (DNA) damage response and cell differentiation [47,122]. In uveal melanoma deletions of chromosome 3 and BAP1, inactivating mutations lead to loss of BAP1 expression and as a consequence, BAP1 mutant cells exhibit an upregulated gene set of OXPHOS [47] and thus mitochondrial metabolism reprogramming [44]. Furthermore, BAP1 mutant samples present metabolic heterogeneity such as either increased glycolysis and nucleotide biosynthesis (OXPHOS-high according to Han A. et al.) or high dependency on fatty acid (FA) oxidation (FAO, OXPHOS-slow) [47]. Han A. et al. highlighted that these different subtypes display differential sensitivity to metabolic inhibitors and as such could influence therapeutic response [47]. Han A. et al. reported 66% of metabolites related to nucleotide biosynthesis pathways upgraded in OXPHOS-high and 64% of fatty acid metabolites increased in OXPHOS-slow (34% increased FA metabolites versus BAP1 wild-type cell line) [47].

The increase in glucose utilization by BAP1 OXPHOS-high variants has been linked by Han. A. et al. to upregulation of Glucose Transporter 3 (GLUT3) and Hexokinase 1 (HK1) and enzymes Glucose-6-Phospate Dehydrogenase (G6PD) and Transketolase (TKT) [47]. Glucose Transporter 1 and 4 (GLUT 1 and GLUT 4) expression remained unchanged [47]. The authors concluded that BAP1 OXPHOS-high mutant cells (MP65 and MP38 cell lines) depend on glycolysis and nucleotide biosynthesis pathways, potentially opening up a metabolic vulnerability of these cell types to pathway inhibitors [47]; interestingly this effect is partially blunted upon re-expressing BAP1 [47]. In contrast, BAP1 OXPHOS-low cells are potentially vulnerable to FAO inhibition via selective inhibition of the mitochondrial ETC Complex I via the IACS-010759 molecule [47,123].

Clinically, BAP1 deletion in uveal melanoma cells induces stem-like features and loss of differentiation [48,124], methylation repatterning [49,124] and transcriptome changes, potentially implicating BAP1 in transcriptional regulatory events that contribute to oncogenic progression and metastasis [50,124].

#### 3.9.1. BAP1 and Ferroptosis (Programmed Cell Death via Lipid Peroxides Accumulation)

The liver is the principal site (90% of cases) of uveal melanoma metastasis [51]. The liver offers a lipid-rich environment as it is the primary organ for storage and metabolism of fatty acids [52]. High bioavailability of lipids in the liver environment is important for uveal melanoma metastases as polyunsaturated fatty acid metabolism is closely associated with ferroptosis, a form of regulated cell death by the accumulation of lipid peroxides [52,125].

Cunanan C.J. et al. investigated the hypothesis that BAP1 loss contributes to the successful metastasis of uveal melanoma through unique management of the lipid-rich liver microenvironment [52]. BAP1 mutant UM cells exhibited consistent enrichment of lipid-related pathways associated with fatty acid metabolism [52] and the authors reported that mutant BAP1 cells proved less sensitive to oxidative stress via higher lipid peroxide levels and thus more resistant to lipotoxicity [52]. Experimental inhibition of lipid metabolism (via atorvastatin) significantly reduced cell proliferation for BAP1 mutant UM cells [52]. BAP1 modulates lipid metabolism via the BAP1 and Additional Sex Combs Like-2 (ASXL2) [52,126,127,128]. The complex formed by BAP1/ASXL2 regulates protein deubiquitination, particularly against histone H2A and reduction of BAP1 correlates with increased histone H2A ubiquitination [52]. Previously in this paper we noted that metastatic uveal melanoma heavily relies on mitochondrial oxidative phosphorylation via OXPHOS [104] via the macroH2A1 histone variant [104] and loss of macroH2A1 decreases mitochondrial metabolism and reduces the aggressivity of UM [43]. According to Cununan C.J. et al., the BAP1/ASXL2 axis appears to influence Peroxisome Proliferator-Activated Receptors (PPARs) expression [52] as ASXL2 depletion reduces PPAR levels in BAP1-competent UM cells [52]. Cununan C.J. et al. suggested potential therapeutic implications such as augmenting Tebentafusp with agents that induce lipid-peroxidation ferroptotic cell death (such as atorvastatin) [52]. Cununan C.J. et al. remarked that Tebentafusp is a bispecific T-cell engager that recruits CD3+ T-cells to GP100-expressing melanoma, and eliciting lipid-peroxide-based cell death in BAP1 mutant UM could be immunogenic and augment Tebentafusp activity [52].

#### 3.9.2. BAP1 and Lactate Metabolism

In cutaneous melanoma, BAP1 mutations promote tumor cell glycolysis leading to increased lactate production [129]. According to Han A. et al., BAP1 mutant UM tumors have an elevated glycolytic gene expression signature [53]. Zheng G. et al. highlighted that BAP1 interacts directly with lactate dehydrogenase (LDHA), causing LDHA to accumulate in the nucleus [129]. Deletion of BAP1 in cutaneous melanoma causes LDHA to accumulate in the cytoplasm instead of the nucleus, catalyzing production of lactate from pyruvate and increasing intracellular and extracellular lactate levels [129]. To our knowledge no papers directly explore the BAP1-LDHA-axis in uveal melanoma and more connecting data is needed.

Han A. et al. highlighted a link between BAP1, pyruvate dehydrogenase (PDH) inactivation and glycolysis in uveal melanoma [53]. The Pyruvate Dehydrogenase Complex (PDC) is composed of Pyruvate Dehydrogenase (PDH, E_1_), Dihydrolipoamide Acetyltransferase (E_2_, DLAT) and Dihydrolipoamide Dehydrogenase Subunit (E_3_, DLD) [53,130]. The PDC converts pyruvate to Acetyl Coenzyme A (Acetyl-CoA) and is reversibly controlled through phosphorylation of PDH by PDH Kinases (PDHKs) and PDH Phosphatases (PDPs) [53,130]. PDH Phosphorylation can be inactivated by multiple allosteric regulators (ATP/ADP ratio) [53,130,131]. Han A. et al. highlighted that PDH inactivation and/or overexpression increases glycolysis in cancer cells and that this behavior is found in uveal melanoma, where BAP1 mutant UM tumors present elevated glycolytic gene signature and high PDC and PDHK1 gene expressions versus BAP1 wild-type UM tumors [53]. Furthermore, suppression of PDH phosphorylation significantly affects BAP1 mutant UM cells, decreasing cell growth and the glycolytic abilities of only BAP1 mutant cells and not wild-type UM cells [53]. Overall, BAP1 mutant UM cells have both increased total and phosphorylated PDH [131]. O-GlcNAcylation of phosphoglycerate kinase 1 leads its translocation to the mitochondria, resulting in PDHK1-PDH phosphorylation and elevation of glucose usage for tumoral growth [131,132]. According to Nie H. et al., coordination of glycolysis and mitochondrial metabolism through regulation of PGK1 glycosylation can promote cell proliferation and tumorigenesis [132] with O-GlcNAcylation regulating Phosphoglycerate Kinase 1 (PGK1), the first ATP-generating enzyme in glycolysis [132].

The principal redox and metabolic characteristics of uveal melanoma discussed above are summarized in Table 2.

### 3.10. Redox Metabolism Parallels and Differences in Uveal Melanoma vs. Other Cancers

#### 3.10.1. Oxidative Phosphorylation (OXPHOS) Pattern

As previously mentioned, metastatic uveal melanoma heavily relies on mitochondrial oxidative phosphorylation (OXPHOS) [104], with tumors exhibiting OXPHOS-high and OXPHOS-low variants [47]. OXPHOS metabolism is also involved in other aggressive and/or treatment-resistant metastatic cancers:

Small cell lung carcinoma (SLCL) cancer cells of SCLC-A molecular subtype feature the Achaete-Scute Homolog 1 (ASCL-1) gene expression, which acts as a driver for a particular metabolic phenotype which is shifted towards increased OXPHOS [133]. According to Solta A. et al., SCLC-A presents significant upregulation of proteins Cytochrome C Oxidase Subunit 4 Isoform 1 (COX4I1), Cytochrome C Oxidase Subunit 5B (COX5B) and NADH Dehydrogenase (Ubiquinone) 1 Alpha Subcomplex 5 (NDUFA5) [133]; these were uniformly upregulated in SCLC-A [133]. Other mutations in mitochondrial proteins involved in the electron transport chain and controlling mitochondrial dynamics were also encountered but not uniformly distributed [133]. The authors concluded that CLC-A is associated with higher mitochondrial content (number of mitochondria) as well as a particular mitochondrial aspect of elongated mitochondria. Furthermore, OXPHOS-high SLCL cells presented less fatty acid metabolism versus OXPHOS-low cells [133].

Pancreatic cancer cells that exhibit oncogene ablation resistance are dependent on OXPHOS [134]. Viale, A et al. concluded that surviving pancreatic cancer cells responsible for tumor relapse present stem-cell-like features and metabolism reliant on OXPHOS [134], with prominent expression of genes governing mitochondrial function, autophagy, lysosome activity and strong reliance on OXPHOS mitochondrial respiration in lieu of dependence on glycolysis [134]. The study noted the high sensitivity of these pancreatic cancer cells to OXPHOS inhibitors, a potential therapeutic target (together with the Kirsten Rat Sarcoma Virus oncogene K-Ras) for inhibiting tumor recurrence [134].

A fraction of metastatic breast cancer populations resistant to estrogen receptor treatment and Cyclin-Dependent Kinases 4 and 6 (CDK4/6) inhibitors exhibit phenotypes highly reliant on OXPHOS [135] and are susceptible to OXPHOS inhibitor IACS-010759 [135], which impairs fatty acid β-oxidation. Levels of glutathione, an antioxidant system, were greater in treatment-resistant cells, and inhibition of glutathione synthesis further made cells more vulnerable to OXPHOS inhibitor IACS [135]. Shen X. et al. also noted that chemotherapy-resistant breast cancer cells exhibit increased mitochondrial OXPHOS and found that metabolic targeting of OXPHOS enhances the chemosensitivity of triple-negative breast cancer cells (resistant to estrogen receptors, progesterone receptors and Human Epidermal Growth Factor Receptor 2 (HER2) HER2-protein-targeted therapy) [136].

Acute myeloid leukemia stem cells (LSCs) are uniquely reliant on OXPHOS, which is maintained by the anti-apoptotic protein B-cell Lymphoma 2 (BCL-2) [137]. BCL-2 inhibition particularly targets OXPHOS [138]. Lagadinou et al. noticed that cancer populations that depend on glycolysis for energy generation secrete lactate as a byproduct that can be onwards used as fuel for OXPHOS [138] and that quiescent LSC subsets may benefit in long-term survival from using OXPHOS to compensate for limited nutrients in the tumoral microenvironment [138].

Cutaneous melanoma exhibits varied metabolic plasticity, with simultaneous upregulation of both OXPHOS and glycolysis vital for melanoma progression and resistance to treatment [139,140]. The metabolic phenotypes display heterogeneity and plasticity between glycolysis and OXPHOS, conferring survival advantages [139]. The B-Raf Proto-Oncogene (BRAF) mutations regulate, via Peroxisome Proliferator-Activated Receptor Gamma Coactivator 1-alpha (PGC1α) and the Melanocyte Lineage Factor (MITF), metabolic reprogramming of cutaneous melanoma towards oxidative metabolism (OXPHOS) [141]. BRAF, most commonly the BRAFV600E mutation, can also regulate the BRAF/MEK/ERK (MAPK) pathway [142] (Mitogen-activated protein kinase kinase MEK, also known as MAP2K or MAPKK and Extracellular Signal-Regulated Kinase ERK). Castellani G. et al. noted that BRAF V600E mediates immune escape in cutaneous melanoma through several mechanisms [142]: release of immunosuppressive mediators such as IL-6 in the tumoral microenvironment (TME), influencing the composition and phenotype of nearby cells, downregulation of melanoma differentiation antigen expression and downregulation of Major Histocompatibility Complex (MHC) [142]. Finally, for the glycolytic metabolism pathway, Kumar et al. highlighted that B-Raf activation of the (MAPK) pathway [139] promotes hypoxia-inducible factor 1α (HIF1α) resulting in increased glycolysis (HIF1α acts as a master regulator of glycolysis) [139]. Interestingly, through inhibiting Microphthalmia-associated Transcription Factor (MITF) and Peroxisome Proliferator-activated Receptor-γ coactivator 1α (PCG-1α), b-RAF inhibits OXPHOS in cutaneous melanoma [141,143]. Overall, the high metabolic plasticity of cutaneous melanoma [140] is owed, in part, to certain mutant tumor populations switching from glycolysis to OXPHOS mitochondrial metabolism. This switch could have clinical implications since treatment with BRAF and MEK inhibitors, treatment that thus inhibits aerobic glycolysis, is not enough for the sub-population of OXPHOS cells that would survive these inhibitors [144].

Prostate cancer cells seem to switch from glycolytic metabolism of normal prostate cells to OXPHOS metabolism, consuming citrate to power OXPHOS or fuel lipogenesis [145]. A high level of pyruvate could favor mitochondrial OXPHOS, whereas a moderate level would favor glycolysis [145]. Chen, C. L. et al. highlighted a regulatory circuit of pyruvate in prostate cancer cells dependent on the final and rate-limiting glycolysis enzyme Pyruvate kinase muscle isozyme 2 (PKM2), Pyruvate transport into mitochondria by Mitochondrial Pyruvate Carrier (MPC) and pyruvate gatekeeping-controller Pyruvate Dehydrogenase Complex (PDC), Lactate Dehydrogenase (LDH) that catalyzes the conversion between pyruvate and lactate, Isocitrate dehydrogenase (IDH) that catalyzes the oxidative decarboxylation of isocitrate to produce α-ketoglutarate (α-KG) and carbon dioxide (CO_2_), and Sirtuins (SIRTs), NAD+-dependent histone deacetylases (HDACs) that can increase mitochondria activity and energy efficacy and reduce damaged ROS [145]. Mutations in mitochondrial DNA (10% of prostate cancer patients) in the Cytochrome Oxidase Subunit I (COI) increased ROS production and tumor growth, and have been associated with drug resistance [145,146]. Schöpf, B. et al. reported a metabolic shift from glutamat/malate (Complex I)-driven OXPHOS in benign tissue to succinate (Complex II)-driven OXPHOS in high-grade prostate cancer [146,147]. Sun, Q. discussed that several OXPHOS Complex I inhibitors that might be suitable for prostate cancer are undergoing clinical trials [147].

In metastatic renal cell carcinoma (RCC), OXPHOS phenotypes are a risk factor for resistance to immune checkpoint inhibitor (ICI) treatment [148]. Translocation renal cell carcinoma (tRCC) is an agressive subtype of kidney cancer that is driven by Transcription Factor Binding to IGHM (TFE3) (IGHM, Immunoglobulin Heavy Chain Mu Enhancer Region) gene fusions [149] that rewrite tRCC towards OXPHOS, in contrast to the glycolytic nature of other kydney cancer types. Impairments in OXPHOS in RCC may also aid immune escape of the primary tumor and epithelial-to-mesenchymal transition (EMT), which may be important for early metastasis [150] (Arner E.N. et. al. [150], PrePrints/not peer-reviewed paper, please see references for more detail). Impairment of OXPHOS is also a key feature of clear cell renal cell carcinoma (ccRCC) [151].

In primary glioblastoma, the messenger ribonucleid acid (mRNA) mRNA-binding oncofetal protein, Insuline-like Growth Factor 2 (IMP2), controls OXPHOS [152] by binding several mRNAs that encode mitochondrial respiratory complex subunits and interaction with Complex I (NADH:ubiquinone oexidoreductase) [152].

In ovarian cancer, OXPHOS variants [153] present only mild response to glucose or glutamine withdrawal and exhibit growth in the abscence of glucose in the TME [154].

In summary, according to Qiu, X et al., crosstalk between oxidative phosphorylation and immune escape in cancer could provide therapeutic leverage [153]. Zhao Z. et al. concluded that OXPHOS is an important metabolic process that drives drug-resistance and OXPHOS targeting could provide novel therapeutic avenues [155].

#### 3.10.2. Glycolysis and Lactate Production Pattern

Most solid tumors that are dependent on glycolysis for energy thrive in a more acidic tumoral environment (i.e., the Warburg effect, intense glycolysis produces lactate that lowers TME pH) [156]. Longhitano L. et al. showed that, for uveal melanoma, accumulation of lactate inhibits cell growth, increases euchromatin rate and enlarges nuclei—features compliant with cell quiescence [6]. The UM cells in turn respond to lactate supplementation in the TME by increasing mRNA expression levels for genes encoding mitochondrial activity, shifting the UM cell metabolism towards oxidative phosphorylation (OXPHOS) [6]. Upon lactate treatment, mRNA levels of PPARG coactivator 1 alpha (PGC1a), Sirtuin 1 (SIRT1) and transcription factor A, mitochondrial (TFAM) achieve a four-fold increase [6], which is associated with an increase in ATP synthase (ATPsyn), Cytochrome C Oxidase Subunit 4 (COX IV), Cyclooxigenase-2 (COX II) and Mitochondrial NADH-Ubiquinone Oxidoreductase Chain 4 (ND4). Furthermore, mRNA expression of LDHA and MCT4 also rises [6]. As such, in effect, uveal melanoma presents a hybrid glycolysis/OXPHOS-switchable metabolism that aids tumoral cell survival. Glycolytic metabolism also helps uveal melanoma cells escape immune response by metabolic reprogramming via IL-6-driven lactate production [38].

In BRAF-mutant cutaneous melanoma, oncogenic signaling augments glycolysis and attenuates respiration [18]. As previously mentioned, BRAF, most commonly BRAFV600E, suppresses mitochondrial respiration through the BRAF/MEK/ERK (MAPK) pathway [142] and furthermore BRAF V600E also mediates immune escape in cutaneous melanoma through several mechanisms [142] (release of immunosuppressive mediators such as IL-6 in the TME, influencing the composition and phenotype of nearby cells, downregulation of melanoma differentiation antigen expression and downregulation of MHC [142]). Hypoxia-inducible factor 1α (HIF1α) is promoted by activation of the MAPK pathway [139], resulting in increased glycolysis since HIF1α acts as a master regulator of glycolysis [139]. Peroxisome Proliferator-Activated Receptor Gamma Coactivator 1-alpha (PGC1α), part of a small family of transcriptional coactivators, promotes mitochondrial biogenesis and respiration [157]. According to Vazquez et al., cutaneous melanoma cells with low levels or negative in PGC1α are more glycolytic and sensitive to ROS-inducing drugs [157] and thus exhibit a more pronounced glycolytic “Warburg” state [157].

In breast cancer, tumoral cells tolerate and use lactate at clinically relevant concentrations [158]. In vivo uptake of lactate is faster than uptake of glucose, coinciding with hypoxic tumoral areas [158]. Lactate can be used as a substrate for aerobic cancer cells; high lactate-consumer cell lines showed less reliance on glucose for cell growth versus low lactate-consumers [158]. Lactate is used in breast cancer cells via conversion to pyruvate, mitochondrial metabolism and subsequent OXPHOS [158]. Kennedy K.M. et al. noted that certain tumor cell lines (R3230Ac) can consume both glucose and lactate simultaneously in vivo, converting lactate to alanine and glutamate [158]. Furthermore, tumoral cell symbiosis is possible with lactate produced by hypoxic tumor cells uptaken via MCT transporters by better perfused aerobic tumoral cells [158]. Nearby cells can also produce lactate: tumor-associated fibroblasts undergo aerobic glycolysis, producing lactate to be used as fuel by tumoral cells (the “Reverse Warburg Effect” according to Pavlides S. et al.) [158,159,160,161,162].

In prostate cancer cells, oxidative PCa cells take up lactate via MCT-1 and use it as fuel [163]. Lactate is then converted back to pyruvate by LDHB and metabolized through the TCA cycle and then OXPHOS [163]. Chetta P. et al. concluded that shuttling lactate between glycolytic and oxidative prostate cancer cells or between glycolytic Cancer-Associated Fibroblasts (CAFs) and oxidative prostate cancer cells represents an adaptation for tumoral survival in nutrient- and oxygen-deprived TME [163]. This mechanism is thus similar in function to the above-described mechanism in breast cancer tumoral cells [158,159,160,161,162]. CAFs can also promote an invasive phenotype by directly supplying PCa cells with functional mitochondria via cellular “bridges” (mitochondrial transfer) [163,164].

ASCL1-driven Small Cell Lung Cancer (SLCL), as previously discussed, exhibits metabolic reprogramming [133]. Ding. X. et al. reported that suppressing lactate production in SLCLA via targeting Phosphoglycerate Kinase 1 (PGK1) and the downstream p-PGK1/p-PDHK axis slows progression of SLCL [165]. As such, lactate inhibition could be a potential therapeutic target in SLCL [165]. Chen J. et al. reported another potential drug target for SLCL in inhibiting lactate via a Monocarboxylate Transporter 1/2 (MCT1/2) inhibitor [166].

In pancreatic ductal adenocarcinoma, Cancer-associated fibroblasts (CAFs) with high levels of glycolysis form an acidic TME that favors survival of pancreatic cancer cells [167]. According to Tan S. et al., pCAFs secrete lactic acid that in turn produces the lactylation of histone H3K18, an epigenetic upregulation that enhances tumoral invasive capacity via the neuroinvasion-associated genes L1CAM (encoding L1 Cell Adhesion Molecule) and SLIT [167,168]. In addition, lactic acid can bind to G-protein-coupled receptor 81 (GPR81) to promote tumor cell energy metabolism, proliferation of cells and metastasis via facilitated transport of lactate [167]. Finally, lactylation has an effect on chemoresistance since it enhances MRN gene repair complex (a protein trio of Mre11, Rad50 and Nbs1) activity which counteracts DNA damage from chemotherapy [167].

In colorectal cancer, enhanced glycolysis is associated with chemoresistance (via the YAP1/GLUT pathway, Yes-1 Associated Transcriptional Regulator and Glucose Transporter family of membrane proteins) [169]. YAP promotes proliferation and migration of colorectal cancer cells via the GLUT3/Adenosine 5′-monophosphate (AMP)-activated protein kinase signaling pathway [169,170]. Research is ongoing into glycolysis inhibitors targeting pyruvate kinases [169]. LDHA induces chemoresistance by promoting biosynthesis and glycolysis and is involved in cancer invasion and the cancer stem cell (CSC) phenotype through the acidic TME maintained by lactic acid output [169,171].

Renal cell carcinoma presents increased glucose uptake and utilization [172]. One of the pathways for glycolysis is via Glucose-6-phosphate dehydrogenase (G6PDH), the rate-limiting enzyme of the pentose phosphate pathway. Furthermore, Lucarelli G. et al. added increased expression of Transketolase (TKT) and higher levels of Pentose Phosphate Pathway (PPP)-derived metabolites (metabolites derived from the aforementioned G6PDH and TKT) [172]. The Monocarboxylate transporter MCT4 plays an important role in lactate metabolism of clear cell renal cell carcinoma (ccRCC), whereas silencing MCT4 results in intracellular acidosis, reduction of intracellular ATP production and partial reversion of the Warburg effect [173]. Although ccRCC is mainly glycolytic in metabolic behavior, metastatic lesions can regain OXPHOS activity [148], which generates resistance to immune-checkpoint therapy [148].

In glioblastoma, expression of hypoxia and glycolytic markers, as well as MCT1 and MCT4 isoforms, varies due to high cellular heterogeneity, with necrotic regions associated with hypoxia [174]. Hypoxia increases plasma membrane expression of MCT1, Cluster of Differentiation 147 (CD147, a transmembrane glycoprotein) and GLUT-1 glutamate transporter, as well as cytoplasmic Hexokinase-2 (HKII) expression [174].

Table 3 summarizes redox and metabolic correlations between uveal melanoma and other cancers.

## 4. Discussion

### 4.1. Hybrid or Symbiosis Glycolysis/OXPHOS Metabolism in Uveal Melanoma?

According to Devic S. et al., fully oxidating one molecule of glucose can produce, in ideal cellular conditions, 38 molecules of ATP to be used as fuel for the cell [175]. In the first step of glycolysis in the cytoplasm, two ATP and two pyruvic acid molecules are produced [175]. If there is sufficient oxygen, pyruvic acid gets converted to Acetyl-Coenzyme A, which onwards enters the citric acid cycle (Krebs cycle) within the mitochondria, followed by participation in the electron transport chain process on the inner mitochondrial membrane [175]. The last step in the electron transport chain requires oxygen to collect the terminal electron from the last cytochrome (Cytochrome A3), which becomes a nascent O^−^ which collects 2H^+^ and produces as a byproduct one water molecule [175]. The previously mentioned oxygen-sufficient path, Aerobic glycolysis, creates the most ATP molecules per molecule of glucose. Anaerobic glycolysis (anaerobic cellular respiration) is performed by the cell under hypoxic conditions: pyruvic acid is converted to lactic acid instead of acetyl-coenzyme A and at the end of the process only two ATP molecules are further produced; thus, anaerobic glucose metabolism is highly ineffective versus aerobic glycolysis [175]. In the tumoral microenvironment (TME), the high growth rate of cancerous cells can outstrip available oxygen supply as can the angiogenesis rate [175]. In consequence, tumoral cells that can perform metabolic reprogramming potentially have better odds of survival and multiplication, effectively outcompeting tumoral populations that have less metabolic plasticity.

In the classic Warburg effect, found to be characteristic of aggressive cancers, tumoral cells perform the conversion of glucose to lactic acid despite the presence of sufficient oxygen [175]; thus, these cancer cells prefer an ineffective metabolic pathway that only produces two ATP molecules per one glucose molecule as opposed to 38 ATP molecules; however, this path does produce twice the lactic acid molecules per glucose molecule: four lactic acid molecules versus only two in normal cell aerobic glycolysis [175]. Lactate production has been found to be pivotal in molding the tumor microenvironment: impacting cell signaling pathways, augmenting the lactate shuttle process, boosting resistance to oxidative stress [166]. Lactylation is a post-translational modification that affects histone and non-histone proteins, influencing a variety of cellular processes, accelerating tumor onset, proliferation, metastasis and the development of drug resistance [166]. Lactate can enter cells through various pathways, such as intercellular transport via MCT (a process known as metabolic symbiosis) [166]; in uveal melanoma, Longhitano L. et al. noted that lactate supplementation boosts the expression of the transporters, and boosts Oxidative Phosphorylation (OXPHOS) activity [6]. All of the above may be advantageous to the selection and survival of highly metabolically plastic tumoral cells in an increasingly acid tumoral microenvironment due to lactic acid production.

According to Onken M.D. et al., in uveal melanoma, oncogenic Gq/11 signaling (from GNAQ and GNA11 oncogenes) drives both glycolysis and respiration [18]. This is in stark contrast to the classic Warburg effect known for other tumors including BRAF-driven cutaneous melanoma [18]. As summarized by Longhitano L. et al., when lactate accumulates enough to inhibit uveal melanoma cell growth and produce cell quiescence [48], uveal melanoma cell populations respond by metabolically reprogramming by increasing mRNA expression levels for genes encoding mitochondrial activity, shifting the UM cell metabolism towards oxidative phosphorylation (OXPHOS) [6]: mRNA levels of PPARG coactivator 1 alpha (PGC1a), Sirtuin 1 (SIRT1) and transcription factor A, mitochondrial (TFAM) achieve a four-fold increase [6], which is associated with an increase in ATP synthase (ATPsyn), Cytochrome C Oxidase Subunit 4 (COX IV), Cyclooxigenase-2 (COX II) and Mitochondrial NADH-Ubiquinone Oxidoreductase Chain 4 (ND4) [6]. A secondary effect of lactate production is the immune-escape phenomenon, which is mainly mediated via IL-6 in uveal melanoma [6]. IL-6 exposure led to upregulation in Programmed Cell Death Ligand 1 (PD-L1) and Human Leukocyte Antigen E (HLA-E) levels, thus favoring immune escape [38]. Furthermore, IL-6 increased enzyme activity encoding Phosphofructokinase (PFK) and Pyruvate Kinase (PKM) in the glycolysis pathway, increased production of lactate and suppressed enzymes in the acetyl-CoA synthesis pathway [38]. Thus, in effect, metastatic or aggressive forms of uveal melanoma present high metabolic plasticity, which has dual effects: favors tumoral survival in both oxygen-rich and hypoxia conditions and promotes metastasis via the immune-escape phenomenon.

### 4.2. Metabolic Targeting in Uveal Melanoma—A Future Therapy Avenue for Aggressive or Metastatic Uveal Melanoma?

Uveal melanoma is clinically particular in that despite effective primary tumor treatment, one third of metastases develop after 5 years [176]; in total, Pereira P.M. et al. noted that approximately 50% of patients develop metastatic disease years later after primary tumor treatment [176]; Rantala E.S. et al. noted that 52% of uveal melanoma results in clinical metastases by 35 years after primary tumor treatment and in 90% of cases the liver is the first metastasis site [177].

According to Rantala E.S. et al., the final frontier in conquering uveal melanoma lies in improving treatment for metastasis disease [177]. Unfortunately, uveal melanoma is genetically distinct from cutaneous melanoma and, as such, common systemic agents like dacarbazine, cisplatin, treosulfan, fotemustine, temozolomide [178], as well as immunomodulatory treatment via T-Lymphocyte-Associated Antigen 4 (CTL4) monoclonal antibody agonist Ipilimumab and Programmed-Cell-Death-1 (PD1) inhibitors Nivolumab and Pembrolizumab [178] are not as effective in uveal melanoma as in cutaneous melanoma [178].

Metabolic targeting could be a future therapeutic avenue in uveal melanoma. These drugs would theoretically target metabolic vulnerabilities characteristic of tumoral sub-populations in malign uveal melanoma.

#### 4.2.1. OXPHOS Metabolic Targeting in Uveal Melanoma

BAP1 mutations are associated with metastasis in uveal melanoma [47]. Transcriptomic analysis of BAP1 shows OXPHOS-high and OXPHOS-low subgroups [47]. OXPHOS-high mutant cells depend on glycolysis and nucleotide biosynthesis pathways [47]. OXPHOS targeting could offer potential novel therapeutic avenues for uveal melanoma.

Teh J.L.F. et al. reported that IACS-010759, an OXPHOS inhibitor, in combination with a Mitogen-Activated Protein Kinase (MEK) inhibitor and a Cyclin-Dependent Kinase 4 and 6 inhibitor (CDK4/6i) decreased UM cell survival [179].

Chattopadhyay, C. et al. studies the use of imipridones (molecules ONC212 and ONC201), indirect OXPHOS inhibitors that activate the Caseinolytic Mitochondrial Matrix Peptidase Proteolytic Subunit (CLPP) [180]. Activation of CLPP leads to dysregulation in proteolysis of mitochondrial OXPHOS effectors Succinate Dehydrogenase Complex Flavoprotein Subunit A (SDHA), Succinate Dehydrogenase Complex Iron-Sulfur Subunit B (SDHB) and NADH Dehydrogenase (Ubiquinone) Fe-S Association Factor 12 (also known as NADH Dehydrogenase (Ubiquinone) 1-α Subcomplex Subunit 12) (NDUFA12) [180]. Chattopadhyay, C reported heterogeneous outcomes and efficacy in reducing UM tumor growth via pirimidone ONC212 [180].

Another recent study by Ali M.M. et al. described the imipridone molecule ONC206 as a mechanistically distinct agent in targeting the mitochondrial metabolism of metastatic uveal melanoma [181]. Treatment via ONC206 suppressed OXPHOS activity, downregulated OXPHOS effector proteins and metabolites and induced apoptosis and/or autophagy in human UM cell lines [181]. ONC206 has an effect via reducing ADHA and SDHB levels, altering 42 metabolites, and dose-dependently decreasing nucleotide phosphates, glycolytic intermediates and TCA cycle components such as fumarate linked to Complex II (SDHA/SDHB) activity [181]. In continuation, ONC 206 treatment raised levels of amino acid derivatives and organic acids, suggesting metabolic stress responses and activation of alternative energy production pathways like shifting towards fatty acid oxidation via perceived increases in lipid metabolism intermediates like acetylcarnitine [181]. According to Ali M.M. et al., the UM cells reponded by reprogramming lipid metabolism and polyunsaturated fatty acids (PUFAs) accumulation, with the authors suggesting that since PUFAs are highly susceptible to oxidative damage and PUFA oxidation products induce lipid peroxidation-mediated cell death, targeting accumulated PUFAs after imipiridone ONC206 treatment could open up another metabolic vurnerability of UM cells [181].

#### 4.2.2. GNAQ/11 (Gq.11) Metabolic Effector Targeting in Uveal Melanoma

Onken M.D. et al. reported that inhibition of the oncogenic Gq.11 with the small molecule FR900359 attenuated glucose uptake by UM cells in vivo and in vitro, blunted glycolysis and mitochondrial respiration and reduced levels of several glycolytic and tricarboxylic acid cycle intermediates [18].

Selectively targeting GNAQ/11 activity by increasing reactive oxygen species (ROS) production, such as described by Li Y. et al. using the copper ionophore elesclomol, leads to a signaling cascade that suppresses the migration of UM cells [24]. Li Y. et al. described the following effect cascade [46]: elesclomol transports copper to mitochondria which produce a large amount of ROS by reduction of Cu^2^ to Cu^1^ in GNAQ/11 mutant cells [46], selectively activating LATS1 kinase which promotes YAP phosphorylation in the hippo pathway [46,120], inhibiting its nuclear accumulation which in turn suppresses the migration of uveal melanoma cells [46]. Elesclomol is sinergic with binimetinib, a MEK inhibitor [46].

#### 4.2.3. Disruption of Hypoxia-Inducible Factor

Disruption of hypoxia transcription factors has also been studied in cutaneous melanoma, where hypoxia drives stabilization and translocation to the nucleus of Hypoxia-Inducible Factor 1-Alpha (HIF-1α) and Hypoxia-Inducible Factor 2-Alpha (HIF-2α), interacting with transcriptional coactivators CREB-binding protein (CBP) and p300 that bind hypoxia-responsive elements (HREs) in the promoter region of more than 300 target genes [182]. In cutaneous melanoma, the master proteins involved in melanoma development (MITF and SRY-box Transcription Factor 10 SOX10) are HIF-2α-binding proteins and, as such, HIF-2α inhibition could have increased potential [182]; HIF-1α and HIF-2α drive vasculogenic mimicry in cutaneous melanoma cells (inhibition completely stops melanoma capillary-like structure formation) [182].

Giatromanolaki, A. et al. reported the upregulation of HIF-1α and HIF-2α and their target genes, VEGF and LDHA, in uveal melanoma after hypoxia exposure of UM cells [182,183]. HIF-2α was linked with VEGF expression, Vascular Endothelial Growth Factor 2 Receptor/Kinase Insert Domain Receptor (VEGFR2/KDR) phosphorylation/activation and Lactate Dehydrogenase 5 (LDH5) expression [182,183] and the VEGF/pVEGFR2 autocrine loop and glycolytic enzymes such as LDH5 [182,183]. HIF-1α regulation by microRNA has been evidenced by Xia Z. et al. via promotion of HIF-1α by miR-652 via repression of Homeobox A9 (HOXA9) [182,184], a negative regulator of HIF-1 expression in cutaneous squamous cell carcinoma [182,185]. Dong, L. et al. reported that disruption via Arylsulfonamide 64B of HIF-1α recruitment of p300/CBP cofactors could impair tumoral hypoxia adaptation as well as oncogenic signaling via the CXCR4 and c-Met receptor tyrosine kinase which can bind hepatocyte growth factor (HGF) [35]. Systemic administration of Arylsulfonamide 64B in an orthotopic mouse model suppressed UM eye growth by 70% and reduced the rate of liver metastasis by 50% [35].

### 4.3. Metabolic Reprogramming and Disease Progression to Metastasis

Rantala E.S. et al. highlighted metastatic uveal melanoma as the final frontier [177]. Metastatic disease can be mitigated by improvement in diagnosis (early and correct diagnosis as highlighted by Engelhard S.B. et al., where the highest rate of malpractice litigation in ocular oncology to uveal melanoma (31.3%) was in connection with missed or delayed diagnosis [186]), by effective primary tumor treatment [187] and by an improved understanding of the metabolic pathways to metastasis. Kuranaga, Y. et al. noted that Monosomy of chromosome 3 (M3) characterizes a subtype of Um associated with mutations in the BRCA-1-associated Protein 1 (BAP1) gene located on chromosome 3p21.1, which encodes a deubiquitinase that serves as a tumor suppressor [156] and that patients with the M3-subtype are at high risk (84%) of metastasis, particularly when chromosome 8q is also amplified [156,188,189,190]. Consequently, BAP1 mutations lead to tumoral heterogeneity and OXPHOS activity elevation, characteristics of metastatic uveal melanoma [47].

Perhaps potential adaptations of the treatment to metabolic particularities of the primary tumor could inhibit the growth of high-risk tumoral varieties such as BAP1-mutated UM cell lines. Han A. et al. concluded that targeting cancer cell metabolism can be a promising therapeutic option for BAP1 mutant UM, especially when tailored to the metabolic heterogeneity present in BAP1 mutant UM [47]. Longhitano, L. et al. highlighted lactate as a metabolite potentially regulating certain UM tumoral cell types [6]. Kuranaga, Y. et al. suggested that targeting metabolic plasticity—either alone or in combination with current treatments—may represent an effective therapeutic strategy for UM [156]. Kuranaga, Y. et al. highlighted that mutated mtDNA in UM cells is often caused by abnormal ROS production, which can contribute to altered mitochondrial function and metabolic reprogramming [156,191,192]; these in turn lead to respiratory chain defects, increased oxidative stress production and genomic instability [156,193,194], which is oncogenetic and promotes metastasis [156]. Proteau, S et al. reported, using a kinome-wide Clustered Regularly Interspaced Short Palindromic Repeats (CRISPR)—CRISPR-associated Protein 9 (CRISPR-Cas9) knockout screen, that the Liver Kinase B1 (LKB1)—Salt-Inducible Kinase 2 (SIK2) LKB1-SIK2 module restrains uveal melanoma tumorigenesis and that removing LKB1 enhances proliferation and survival through SIK2 inhibition and upregulation of sodium/calcium exhanger Solute Carrier Family 8 Member A1 SLC8A1 [25]. Finally, Kuranaga, Y. et al. highlighted the disfunctional regulation of apoptosis in UM, with high Apoptosis-inducing Factor (AIF) carrying a poor prognosis for UM [156,195], and UM anti-apoptosis mechanisms such as upregulation of survivin [156,196,197], an anti-apoptotic protein and inactivation of pro-apoptotic signaling cascades [156,198]. Thus Kuranaga, Y. et al. concluded that targeting select enzymes and signaling pathways involved in UM glycolysis, fatty acid metabolism and mitochondrial dynamics might improve treatment effect and/or overcome treatment resistance [156].

## 5. Conclusions

The main challenge to successful treatment of uveal melanoma is not necessarily the primary tumor; instead, the challenge remains in preventing the appearance of metastatic disease years after primary tumor removal and improving metastatic disease treatment. Uveal melanoma presents particularities in glycolysis, lactate, fatty acid and oxidative phosphorylation (OXPHOS) metabolism that are different from cutaneous melanoma and other cancers. Metabolic plasticity of uveal melanoma cells seems to represent one of the fundamental mechanisms which allow tumoral progression, metastasis, resistance to oxidative stress, the immune-escape phenomenon and the development of treatment resistance. To this effect, the high metabolic plasticity and resistance of uveal melanoma may produce, via ongoing metabolic research, new therapy targets and opportunity which, combined with current treatments, could increase survival. Furthermore, while uveal melanoma is genetically distinct, there are parallels with enzymatic and metabolic pathways found in multiple other cancers and potential for treatment crossover from other malignancy research. The reverse is also possible, with uveal melanoma OXPHOS research crossing over into other cancer types. Overall, it is clear that metabolic understanding is an increasingly important avenue for understanding and treating uveal melanoma.

## Figures and Tables

**Table 1:** Studies cited in this paper.

Ref. Nr.	Study Authors	Study Title	Year
[4]	Chua V. et al.	Dysregulated GPCR Signaling and Therapeutic Options in Uveal Melanoma	2017
[5]	Decatur C.L. et al.	Driver Mutations in Uveal Melanoma: Associations With Gene Expression Profile and Patient Outcomes	2016
[6]	Longhitano L. et al.	Lactate Rewrites the Metabolic Reprogramming of Uveal Melanoma Cells and Induces Quiescence Phenotype	2023
[7]	Uner O.E. et al.	Estimation of the Timing of BAP1 Mutation in Uveal Melanoma Progression	2021
[8]	Singh N. et al.	Uveal Melanoma in BAP1 Tumor Predisposition Syndrome: Estimation of Risk	2021
[9]	Kilic E. et al.	SF3B1 and EIF1AX Mutations in Uveal Melanoma: A Protective Factor, or Not?	2014
[13]	Li H. et al.	Inhibition of Chemokine Receptor Expression on Uveal Melanomas by CXCR4 siRNA and Its Effect on Uveal Melanoma Liver Metastases	2009
[14]	Li H. et al.	Differential Expression of Chemokine Receptors on Uveal Melanoma Cells and Their Metastases	2008
[18]	Onken M.D. et al.	Oncogenic Gq/11 Signaling Acutely Drives and Chronically Sustains Metabolic Reprogramming in Uveal Melanoma	2022
[20]	Blasi M.A. et al.	Antioxidant Pattern in Uveal Melanocytes and Melanoma Cell Cultures	1999
[21]	Tonelotto V. et al.	1,4-Dihydroxy Quininib Activates Ferroptosis Pathways in Metastatic Uveal Melanoma and Reveals a Novel Prognostic Biomarker Signature	2024
[22]	Chua V. et al.	The AMP-Dependent Kinase Pathway Is Upregulated in BAP1 Mutant Uveal Melanoma	2022
[23]	Deliktas O. et al.	Modulation of AMPK Significantly Alters Uveal Melanoma Tumor Cell Viability	2023
[24]	Ambrosini G. et al.	Inhibition of Mutant GNAQ Signaling in Uveal Melanoma Induces AMPK-Dependent Autophagic Cell Death	2013
[25]	Proteau S. et al.	LKB1-SIK2 Loss Drives Uveal Melanoma Proliferation and Hypersensitivity to SLC8A1 and ROS Inhibition	2023
[26]	Wang J.Z. et al.	UC2288 Decreases the Viability and Metastatic Activity of Human Uveal Melanoma Cells via Activating the AMPK/eIF2/ATF4 ER Stress Axis	2026
[27]	Ho A.L. et al.	Impact of Combined mTOR and MEK Inhibition in Uveal Melanoma Is Driven by Tumor Genotype	2012
[28]	Babchia N. et al.	The PI3K/Akt and mTOR/P70S6K Signaling Pathways in Human Uveal Melanoma Cells: Interaction with B-Raf/ERK	2010
[29]	Amirouchene-Angelozzi N. et al.	Establishment of Novel Cell Lines Recapitulating the Genetic Landscape of Uveal Melanoma and Preclinical Validation of mTOR as a Therapeutic Target	2014
[30]	Amirouchene-Angelozzi N. et al.	The mTOR Inhibitor Everolimus Synergizes with the PI3K Inhibitor GDC0941 to Enhance Anti-Tumor Efficacy in Uveal Melanoma	2016
[31]	Yan F. et al.	Pristimerin-Induced Uveal Melanoma Cell Death via Inhibiting PI3K/Akt/FoxO3a Signaling Pathway	2020
[32]	Yan F. et al.	Elucidating the Role of the FoxO3a Transcription Factor in the IGF-1-Induced Migration and Invasion of Uveal Melanoma Cancer Cells	2016
[33]	Yan F. et al.	Forkhead Box Protein O3 Suppresses Uveal Melanoma Development by Increasing the Expression of BCL2L11 and CDKN1B	2018
[34]	Durante M.A. et al.	Single-Cell Analysis Reveals New Evolutionary Complexity in Uveal Melanoma	2020
[35]	Dong L. et al.	Arylsulfonamide 64B Inhibits Hypoxia/HIF-Induced Expression of c-Met and CXCR4 and Reduces Primary Tumor Growth and Metastasis of Uveal Melanoma	2019
[36]	Bronkhorst I.H. et al.	Effect of Hypoxic Stress on Migration and Characteristics of Monocytes in Uveal Melanoma	2014
[37]	Singh A.D. et al.	Chromosome 3 Status in Uveal Melanoma: A Comparison of Fluorescence In Situ Hybridization and Single-Nucleotide Polymorphism Array	2012
[38]	Gong C. et al.	IL-6-Driven Autocrine Lactate Promotes Immune Escape of Uveal Melanoma	2024
[39]	Robertson A.G. et al.	Integrative Analysis Identifies Four Molecular and Clinical Subsets in Uveal Melanoma	2017
[40]	Wu Y. et al.	Oxidative Stress-Related Genes in Uveal Melanoma: The Role of CALM1 in Modulating Oxidative Stress and Apoptosis and Its Prognostic Significance	2025
[41]	García-Mulero S. et al.	Additive Role of Immune System Infiltration and Angiogenesis in Uveal Melanoma Progression	2021
[42]	Neo S.Y. et al.	Natural Killer Cells Drive 4-1BBL Positive Uveal Melanoma Towards EMT and Metastatic Disease	2024
[43]	Giallongo S. et al.	Loss of macroH2A1 Decreases Mitochondrial Metabolism and Reduces the Aggressiveness of Uveal Melanoma Cells	2020
[44]	Zhan Z. et al.	Construction of Oxidative Phosphorylation-Related Prognostic Risk Score Model in Uveal Melanoma	2024
[45]	Elbatsh A.M.O. et al.	INPP5A Phosphatase Is a Synthetic Lethal Target in GNAQ- and GNA11-Mutant Melanomas	2024
[46]	Li Y. et al.	Copper Ionophore Elesclomol Selectively Targets GNAQ/11-Mutant Uveal Melanoma	2022
[47]	Han A. et al.	BAP1 Mutant Uveal Melanoma Is Stratified by Metabolic Phenotypes with Distinct Vulnerability to Metabolic Inhibitors	2021
[48]	Matatall K.A. et al.	BAP1 Deficiency Causes Loss of Melanocytic Cell Identity in Uveal Melanoma	2013
[49]	Field M.G. et al.	BAP1 Loss Is Associated with DNA Methylomic Repatterning in Highly Aggressive Class 2 Uveal Melanomas	2019
[50]	Karlsson J. et al.	Molecular Profiling of Driver Events in Metastatic Uveal Melanoma	2020
[51]	Huibers A. et al.	Management of Liver Metastases from Uveal Melanoma	2025
[52]	Cunanan C.J. et al.	BAP1 Loss Affords Lipotoxicity Resistance in Uveal Melanoma	2025
[53]	Han A. et al.	Pyruvate Dehydrogenase Inactivation Causes Glycolytic Phenotype in BAP1 Mutant Uveal Melanoma	2022

Studies are presented in the order of appearance in the review. Ref. nr.—Reference number.

**Table 2:** Principal redox and metabolic characteristics of uveal melanoma.

Uveal Melanoma Redox Metabolic Characteristics		
Metabolic Characteristic	Genetic Mutations or Target Molecules/Receptors	Effect
Genetic heterogeneity of tumoral populations	EIF1AX mutation in class 1A, SD3B1 and other splicing mutations in class 1B, BAP1 mutations in class 2 [34]	Advantages in eluding immune surveillance Creates immunosuppressive environment
Hypoxia resistance and adaptation	HIF activation leading to gene expression, expression of proinflammatory cytokines PLGF, TGFβ, END1, ICAM1 [35,36]	Changes host immune response to favor immunosuppressive and proangiogenic M2 macrophages [36]
Metabolic reprogramming from lactate accumulation	Lactate switches metabolism from glycolysis towards OXPHOS [6].	Slows down glycolytic activity [6]. Switches metabolism towards OXPHOS [6].
	Lactate increases IL-6 secretion, upregulating PD-L1 and HLA-E levels and favoring immune escape [38,39,101].	Favors immune-escape via upregulated IL-6 secretion [38,39,101].
	Lactate accumulation can create more lactate via IL-6 in a feed-forward loop for increasing lactate concentrations	Favors the creation of more lactate via feed-forward loop lactate -> IL-6 -> PFK and PKM enzymes -> more lactate [38,39,101]
Resistance to oxidative stress	CALM1 overexpression [40]	oeCALM1 effectively reverses measured oxidative stress by increasing SOD and suppressing accumulation of MDA and LDH [40].
	CALM1 can influence CD4+ T-Cell response via regulation of Ca^2+^ signaling, affecting D4+ T-cell activation and naive CD4+ T-cells differentiation [40]	CALM1 via Ca^2+^ signaling affects immune response by modulating CD4+ T-cell activation and naive CD4+ T-cells differentiation [40]
	Calcium-ROS axis. ROS interact with Ca^2+^ signaling, regulating cellular Ca^2+^ [103]	Chain effects on endoplasmic reticulum, cytosol, nucleus, mitochondria, lysosomes and plasma membrane [103]
High capability for OXPHOS metabolism	Metastatic uveal melanoma heavily relies on OXPHOS via the macroH2A1 histone variant [104].	OXPHOS confers metabolic resilience in hypoxic environments [6,104]
	Zhan Z. et al. 9 OXPHOS prognostic genes: NDUFB1, NDUFB8, ATP12A, NDUFA3, CYC1, COX6B1, ATP6V1G2, ATP4B, NDUFB4 [44]	OXPHOS activity has mutagenic potential: increased OXPHOS generates electron leakage that reacts with molecular oxygen to generate superoxide and other ROS [106,107]
Oncogene-driven metabolism	GNAQ and GNA11 drive glucose uptake (glycolysis) and mitochondrial respiration [18]	Favors uveal melanoma progression
	and favor maintenance of redox homeostasis [18]	Maintains redox homeostasis
	GNAQ/GNA11 driven anti-apoptosis resistance [118]	Anti-apoptosis resistance
	BAP1 is associated with OXPHOS-high and OXPHOS-slow tumor cells [47]	Promotes loss of cell differentiation and metastasis
	BAP1 enriches lipid-related pathways associated with fatty acid metabolism [51]	Could promote metastasis to lipid rich liver environment [51]

Abbreviations: EIF1AX—Eukaryotic Translation Initiation Factor 1A X-Linked, SF3B1—Splicing Factor 3b Subunit 1, BAP1—BRCA-1-associated protein 1, PLGF—Placental Growth Factor, TGFβ—Transforming Growth Factor Beta, END1—Endothelin-1, ICAM1—Intercellular Adhesion Molecule 1, OXPHOS—Oxidative Phosphorylation, IL6—Interleukin-6, PD-L1—Programmed Cell Death Ligand 1, HLA-E—Human Leukocyte Antigen E, PFK—Phosphofructokinase, PKM—Pyruvate Kinase, CALM1—Calmodulin-1, SOD—Superoxide Dismutase, MDA—Malondialdehyde, LDH—Lactate Dehydrogenase, ROS—reactive oxygen species, GNAQ—Guanine Nucleotide-Binding Protein G(q) subunit α, GNA11—Guanine Nucleotide-binding Protein Subunit α-11.

**Table 3:** Summary of redox and metabolic correlations between uveal melanoma and other cancers.

Oxidative Phosphorylation (OXPHOS)		
Cancer	OXPHOS Pattern	Effect
Uveal melanoma	OXPHOS-high and OXPHOS-slow tumor cells [47]. OXPHOS phenotype driven by BAP1 mutations [47]. OXPHOS-high maintains higher glycolysis and nucleotide biosynthesis pathway; OXPHOS-slow phenotypes have increased fatty acid metabolites.	Tumoral population heterogeneity promotes selection of aggressive tumoral variants within the tumoral microenvironment, promoting survival, mutagenic potential and eventual metastatic disease. OXPHOS metabolism provides resistance to therapeutic agents.
Small cell lung carcinoma (SLCL)	OXPHOS-high and OXPHOS-low cell subtypes ASCL-1 subtype is shifted towards OXPHOS, higher mitochondrial activity [133]	Treatment resistance
Pancreatic Cancer	OXPHOS-reliant tumors present stem-cell-like features, do not depend on glycolysis [134]	Treatment resistance, resistance to oncogene ablation [134]
Metastatic Breast Cancer	OXPHOS-reliant and estrogen-receptor-treatment-resistant tumors [135]	OXPHOS could be a metabolic vulnerability and potential therapeutic target in these estrogen-receptor-treatment-resistant cells
Acute myeloid leukemia stem cells (LSCs)	Uniquely reliant on OXPHOS maintained by anti-apoptotic protein BCL-2 [138]	BCL-2 targeting inhibits OXPHOS and presents a therapeutic target [138]
Cutaneous melanoma	Simultaneous upregulation of both glycolysis and OXPHOS [139,140]. High metabolic heterogeneity. BRAF V600E mutation associated with immune escape [139,140,141,142] and MAPK pathway activation [142]. B-Raf MAPK activation promotes HIF1α, increasingly glycolysis [139]	Metabolic resilience, resistance to therapeutic agents such as BRAF and MEK inhibitors [144], immune escape phenomenon
Prostate cancer	Switch from glycolytic metabolism of normal prostate cells to OXPHOS metabolism in cancerous prostate cells [145]. OXPHOS phenotypes associated with higher-grade prostate cancer [146,147].	OXPHOS linked to higher-grade prostate cancer, metabolic plasticity and treatment resistance
Metastatic renal cell carcinoma (RCC) and translocation RCC (tRCC)	OXPHOS phenotypes are high risk: the aggressive translocation RCC (tRCC) presents extensive metabolic reprogramming from glycolysis to OXPHOS and associated immune escape phenomena [148,149,150,151]	tumoral aggressivity, metastatic potential, immune escape
Ovarian Cancer	OXPHOS variants present only mild response to glucose and glutamine withdrawal and can grow in the absence of sufficient glucose in the TME [153,154]	Tumoral survival in the absence of sufficient glucose in the TME
**Glucose metabolism (Glycolysis) and lactate production**		
Uveal melanoma	GNAQ and GNA11 drive glucose uptake (glycolysis) and mitochondrial respiration [18] Lactate accumulation in the TME drives metabolic reprogramming towards OXPHOS, contrary to the classic Warburg effect [156]	Glycolytic metabolism for tumoral progression; lactate accumulation produces metabolic switch towards OXPHOS, conferring resistance to unfavorable TME for tumoral cell survival, selection of resistant variants and metastasis
Cutaneous melanoma (BRAF and BRAFV600E mutant variants)	Simultaneous upregulation of both glycolysis and OXPHOS [139,140]. High metabolic heterogeneity. The BRAFV600E mutation increases glycolysis via MAPK pathway and HIF1α glycolysis regulation [139,141]. Low levels of PGC1α are more glycolytic and sensitive to ROS-inducing drugs [157].	Tumoral cell survival through high metabolic plasticity.
Breast cancer	Glycolytic metabolism and lactate use as substrate similar to Warburg effect [156,158]. Tumoral symbiosis: hypoxic tumor cells produce lactate that is taken up by better perfused aerobic tumoral cells	Synergy between glycolytic and lactate metabolism. Promotes tumoral growth.
Prostate cancer	Glycolytic/OXPHOS metabolic symbiosis. Lactate shuttle between glycolytic and oxidative cancer cells or between CAFs and oxidative cancer cells [163]	Survival advantage in the TME [163]
Small Cell Lung Cancer (ASCL1 variant)	Lactate plays important role in tumoral proliferation [166].	Lactate targeting is a potential therapeutic target [166]
Pancreatic ductal adenocarcinoma	CAFs secrete lactic acid that lactylates histone H3K18, producing epigenetic upregulations that enhance tumoral invasive capacity [117,118]. Lactylation improves chemoresistance by enhancing MRN gene repair complex, counteracting DNA damage from chemotherapy [167]	Lactate production and lactylation are important potential therapeutic targets
Colorectal cancer	Enhanced glycolysis provides chemoresistance via YAP1/GLUT pathway [169]; LDHA enhances chemoresistance in TME, maintaining lactic acid output [169]. YAP promotes proliferation and migration of colorectal cancer cells [169].	Promotes tumoral cell proliferation and migration, chemoresistance
Renal Cell Carcinoma	Increased glycolysis and Warburg effect [156,172]. ccRCC prefers glycolytic metabolism over OXPHOS.	Glycolytic metabolism preferred. Glycolytic variants sensitive to immune-checkpoint therapy.
Glioblastoma	Very high metabolic heterogeneity [174]. Hypoxic regions are characteristic; hypoxic cells adapt via expression of MCT1, CD147, GLUT-1, HKII [174].	Aggressive tumoral cell growth powered by high metabolic heterogeneity

Abbreviations: OXPHOS—Oxidative Phosphorylation, SLCL—Small cell lung carcinoma, ASCL1—Achaete-Scute Homolog 1, BCL-2—B-cell Lymphoma 2, LSCs—Acute myeloid leukemia stem cells, BRAF—B-Raf Proto-Oncogene, MAPK—Mitogen-Activated Protein Kinase, HIF-1α—Hypoxia-Inducible Factor 1-Alpha, PCG-1α—Peroxisome Proliferator-activated Receptor-γ coactivator 1α, ROS—reactive oxygen species, TME—Tumoral Microenvironment, RCC—Renal Cell Carcinoma, tRCC—Translocation Renal Cell Carcinoma, GNAQ—Guanine Nucleotide-Binding Protein G(q) subunit α, GNA11—Guanine Nucleotide-binding Protein Subunit α-11, CAFs—Cancer-Associated Fibroblasts, SLCL—Small Cell Lung Carcinoma, ASCL1—Achaete-Scute Homolog 1, HKII—Hexokinase-2, DNA—Deoxyribonucleic Acid, YAP—Yes-associated Protein, GLUT1—Glucose Transporter 1, LDHA—Lactate Dehydrogenase, ccRCC—Clear Cell Renal Cell Carcinoma, MCT1—Monocarboxylate Transporter 1, CD147—Cluster of Differentiation 147, GLUT1—Glucose Transporter 1.

## Data Availability

For the purposes of this literature review paper, the presented data pertains to the original individual author’s papers, cited accordingly in the references section.

## Abbreviations

The following abbreviations are used in this manuscript:

UM	Uveal melanoma
CM	Cutaneous melanoma
GPCR	G-protein-coupled receptor
GDP	Guanosine diphosphate
GTP	Guanosine triphosphate
BAP1	BRCA-1-associated protein 1
BAP1-TPDS	BAP1 Tumor predisposition syndrome
SF3B1	Splicing Factor 3b Subunit 1
EIF1AX	Eukaryotic Translation Initiation Factor 1A X-Linked
CXCR4	Chemokine Receptor 4
CCR7	C-C Chemokine Receptor Type 7
ROS	Reactive Oxygen Species
O_2_^•−^	Superoxide Radicals
H_2_O_2_	Hydrogen Peroxide
•OH	Hydroxyl
^1^O_2_	(Singlet) Oxygen
LOX	Lipoxygenase
COX	Cyclooxigenase
SOD	Superoxide dismutase
CAT	Catalase
GPx	Glutathione peroxidase
PRISMA	The Preferred Reporting Items for Systematic Review and Meta-Analyses
WoS	Web of Science
NADPH	Nicotinamide Adenine Dinucleotide Phosphate
NOX1-5	NADPH Oxidases 1 to 5
PUFAs	Polyunsaturated fatty acids
NRF2	Redox-activated Transcription Factor NF-E2-Related Factor 2
KEAP1	Kelch-like ECH-associated Protein 1
TCGA	The Cancer Genome Atlas
MAPK	Mitogen Activated Protein Kinase
EGFR	Epidermal Growth Factor Receptor
SOD1	Cytosolic Superoxide Dismutase
SOD2	Mitochondrial Superoxide Dismutase
SOD3/EC-SOD	Extracellular Superoxide Dismutase
GSH	Glutathione
GPXs	Glutathione Peroxidases
GPX1	Glutathione Peroxidase 1
GPX4	Glutathione Peroxidase 4
GR	Glutathione Reductase
GSSG	Oxidized Glutathione
PRDXs	Peroxiredoxins
PRDX3	Thioredoxin-dependent Peroxide Reductase
PRDX5	Peroxiredoxin 5
TXN	Thioredoxin
TXN2	Thioredoxin 2
TXNRD	Thioredoxin Reductase
TXNRD2	Thioredoxin Reductase 2
LOOH	Lipid hydroperoxides
NADP	Nicotinamide Adenine Dinucleotide Phosphate
IDH2	NADP-Dependent Isocitrate Dehydrogenase
ME3	Malic Enzyme
NNT	Nicotinamide Nucleotide Transhydrogenase
HO-1	Heme Oxygenase 1
NR4A3/NOR1	Nuclear Receptor Subfamily 4 Group A Member 3
AMP	Adenosin monophosphate
ADP	Adenosin diphosphate
ATP	Adenosine triphosphate
ACC	Acetyl-CoA Carboxylase
RNA	Ribonucleic Acid
ER	Endoplasmic Reticulum
mTOR	Mechanistic Target of Rapamycin
mTORC1	Mammalian Target of Rapamycin Complex 1
mTORC2	Mammalian Target of Rapamycin Complex 2
PIKK	PI3K-related kinase
Raptor	Regulatory Protein Associated with mTOR
mLST8/GβL	Mammalian Lethal with Sec13 Protein 8
TOS	TOR signaling
FoxO	Forkhead Box O
IGF-1	Insulin Growth Factor 1
BIM	Bcl-2 Interacting Mediator of Cell Death
PON2	Paraoxonase-2
NMSC	Non-melanoma Skin Cancers
BCC	Basal Cell Carcinoma
siRNA	Small Interfering Ribonucleic Acid
scRNA-seq	Single-cell Ribonucleic Acid Sequencing
HIF	Hypoxia Inducible Factor
HGF	Hepatocyte Growth Factor
PLGF	Placental Growth Factor
VEGF	Vascular Endothelial Growth Factor
TGFβ	Transforming Growth Factor Beta
END1	Endothelin-1
ICAM1	Intercellular Adhesion Molecule 1
AIMP1	Aminoacyl tRNA Synthetase Complex-Interacting Multifunctional Protein 1
CCL2	Chemokine Ligand 2
MCP-1	Chemoattractant Protein-1
IL1β	Interleukin-1-Beta
HCAR1	Hydroxycarboxylic Acid Receptor 1
MCT1	Monocarboxylate Transporter 1
MCT4	Monocarboxylate Transporter 4
OXPHOS	Oxidative Phosphorylation
IL6	Interleukin-6
mRNA	Messenger ribonucleic acid
PD-L1	Programmed Cell Death Ligand 1
HLA-E	Human Leukocyte Antigen E
PFK	Phosphofructokinase
PKM	Pyruvate Kinase
CALM1	Calmodulin-1
MDA	Malondialdehyde
LDH	Lactate Dehydrogenase
CD40L	CD40 Ligand
Th17	T-Helper-17
NK	Natural Killer (Cells)
NDUFB1	Gene encoding NADH Dehydrogenase (Ubiquinone) 1-β Subcomplex Subunit 1
NADH	Nicotinamide Adenine Dinucleotide
NDUFB8	Gene encoding NADH Dehydrogenase (ubiquinone) 1-β Subcomplex Subunit 8
ATP12A	Gene encoding Potassium-transporting ATPase α-chain 2
NDUFA3	Gene encoding NADH dehydrogenase (ubiquinone)
CYC1	The Respiratory Subunit of Ubiquinol Cytochrome C
COX6B1	Gene encoding Cytochrome C Oxidase Subunit 6B1
ATP6V1G2	Gene encoding a Component of Vacuolar ATPase
ATP4B	Gene encoding β-Subunit of the H+/K+-ATPase proton pump
NDUFB4	Gene encoding NADH dehydrogenase (ubiquinone) 1-β-Subcomplex-4
GNAQ	Guanine Nucleotide-Binding Protein G(q) subunit α
GNA11	Guanine Nucleotide-binding Protein Subunit α-11
IP3	Inositol 1,4,5 triphosphate
INPP5A	Inositol-1,4,5-triphosphate 5-phosphatase
LATS1	Large Tumor Suppressor Kinase 1
YAP	Yes-associated Protein
MEK	Mitogen-Activated Protein Kinase
FDA	United States Food and Drug Administration
EMA	European Medicines Agency
BAP1	BRCA1-associated protein
BRCA1	Breast Cancer 1
DUB	Deubiquitinating Enzyme
DNA	Deoxyribonucleic Acid
FA	Fatty Acid
FAO	Fatty Acid Oxidation
GLUT3	Glucose Transporter 3
HK1	Hexokinase 1
GLUT1	Glucose Transporter 1
GLUT4	Glucose Transporter 4
G6PD	Glucose-6-phosphate dehydrogenase
TKT	Transketolase
ASXL	Additional Sex Combs Like-2
PPAR	Peroxisome Proliferator-Activated Receptors
LDHA	Lactate Dehydrogenase A
LDHB	Lactate Dehydrogenase B
PDH, E1	Pyruvate dehydrogenase
PDC	Pyruvate dehydrogenase complex
E2, DLAT	Dihydrolipoamide Acetyltransferase
E3, DLD	Dihydrolipoamide Dehydrogenase Subunit
Acetyl-Coa	Acetyl Coenzyme A
PDHKs	Pyruvate dehydrogenase kinases
PDPs	Pyruvate dehydrogenase phosphatases
PGK1	Phosphoglycerate Kinase 1
SLCL	Small Cell Lung Carcinoma
ASCL1	Achaete-Scute Homolog 1
COX4I1	Cytochrome C Oxidase Subunit 4 Isoform 1
COX5B	Cytochrome C Oxidase Subunit 5B
NDUFA5	NADH Dehydrogenase (Ubiquinone) 1 Alpha Subcomplex 5
K-Ras	Kirsten Rat Sarcoma Virus (Oncogene)
CDK4/6	Cyclin-Dependent Kinases 4 and 6
HER2	Human Epidermal Growth Factor Receptor 2
LSCs	Acute myeloid leukemia stem cells
BCL-2	B-cell Lymphoma 2
MAPK	Mitogen-activated Protein Kinase
PCG-1α	Peroxisome Proliferator-activated Receptor-γ coactivator 1α
BRAF	B-Raf Proto-Oncogene
PGC1α	Peroxisome Proliferator-Activated Receptor Gamma Coactivator 1-alpha
MITF	Melanocyte Lineage Factor or Microphthalmia-associated Transcription Factor (Depending on context)
MEK/MAP2K/MAPKK	Mitogen-Activated Protein Kinase Kinase
ERK	Extracellular Signal-Regulated Kinase
TME	Tumoral Microenvironment
MHC	Major Histocompatibility Complex
PKM2	Pyruvate kinase muscle isozyme 2
MPC	Mitochondrial Pyruvate Carrier
IDH	Isocitrate dehydrogenase
α-KG	α-ketoglutarate
CO_2_	Carbon Dioxide
SIRTs	Sirtuins
HDAC	NAD+-dependent histone deacetylases
COI	Cytochrome Oxidase Subunit I
RCC	Renal Cell Carcinoma
ICI	Immune Checkpoint Inhibitor
Tfe3	Transcription Factor Binding to IGHM
IGHM	Immunoglobulin Heavy Chain Mu Enhancer Region
EMT	Epithelial-to-mesenchymal transition
ccRCC	Clear Cell Renal Cell Carcinoma
PPARG	Peroxisome Proliferator-Activated Receptor Gamma/Peroxisome Proliferator-Activated Receptors
SIRT1	Sirtuin 1
TFAM	Transcription Factor A, Mitochondrial
ATPsyn	ATP synthase
COX IV	Cytochrome C Oxidase Subunit 4
COX II	Cyclooxigenase-2
ND4	Mitochondrial NADH-Ubiquinone Oxidoreductase Chain 4
TCA	Tricarboxylic Acid (Cycle)
CAFs	Cancer-Associated Fibroblasts
GPR81	G-protein-coupled receptor 81
YAP1	Yes-1 Associated Transcriptional Regulator
GLUT	Glucose Transporter (family of membrane proteins)
AMP	Adenosine 5′-monophosphate
CSC	Cancer Stem Cell
G6PDH	Glucose-6-phosphate dehydrogenase
PPP	Pentose Phosphate Pathway
MCT	Monocarboxylate transporter
CD147	Cluster of Differentiation 147
HKII	Hexokinase-2
CLPP	Caseinolytic Mitochondrial Matrix Peptidase Proteolytic Subunit
SDHA	Succinate Dehydrogenase Complex Flavoprotein Subunit A
SDHB	Succinate Dehydrogenase Complex Iron-Sulfur Subunit B
NDUFA12	NADH Dehydrogenase (Ubiquinone) Fe-S Association Factor 12 or known as NADH Dehydrogenase (Ubiquinone) 1-α Subcomplex Subunit 12
HIF-1α	Hypoxia-Inducible Factor 1-Alpha
HIF-2α	Hypoxia-Inducible Factor 2-Alpha
CBP	CREB-binding protein
HRE	Hypoxia-Responsive Elements
SOX10	SRY-box Transcription Factor 10
VEGFR2/KDR	Vascular Endothelial Growth Factor 2 Receptor/Kinase Insert Domain Receptor
LDH5	Lactate Dehydrogenase 5
HOXA9	Homeobox A9
M3	Monosomy of Chromosome 3
CRISPR	Clustered Regularly Interspaced Short Palindromic Repeats
Cas9	CRISPR-associated Protein 9
LKB1	Liver Kinase B1
SIK2	Salt-Inducible Kinase 2
SLC8A1	Solute Carrier Family 8 Member A1
